# ATM pathway activation limits R-loop-associated genomic instability in Werner syndrome cells

**DOI:** 10.1093/nar/gkz025

**Published:** 2019-01-18

**Authors:** Veronica Marabitti, Giorgia Lillo, Eva Malacaria, Valentina Palermo, Massimo Sanchez, Pietro Pichierri, Annapaola Franchitto

**Affiliations:** 1 Department of Environment and Health, Section of Mechanisms Biomarkers and Models, Istituto Superiore di Sanita’, Viale Regina Elena 299, Rome 00161, Italy; 2 Department of Cell Biology and Neurosciences, Section of Gene and Cell Therapy, Istituto Superiore di Sanita’, Viale Regina Elena 299, Rome 00161, Italy

## Abstract

Werner syndrome (WS) is a cancer-prone disease caused by deficiency of Werner protein (WRN). WRN maintains genome integrity by promoting replication-fork stability after various forms of replication stress. Under mild replication stress, WS cells show impaired ATR-mediated CHK1 activation. However, it remains unclear if WS cells elicit other repair pathway. We demonstrate that loss of WRN leads to enhanced ATM phosphorylation upon prolonged exposure to aphidicolin, a specific inhibitor of DNA polymerases, resulting in CHK1 activation. Moreover, we find that loss of WRN sensitises cells to replication-transcription collisions and promotes accumulation of R-loops, which undergo XPG-dependent cleavage responsible for ATM signalling activation. Importantly, we observe that ATM pathway limits chromosomal instability in WS cells. Finally, we prove that, in WS cells, genomic instability enhanced upon chemical inhibition of ATM kinase activity is counteracted by direct or indirect suppression of R-loop formation or by XPG abrogation. Together, these findings suggest a potential role of WRN as regulator of R-loop-associated genomic instability, strengthening the notion that conflicts between replication and transcription can affect DNA replication, leading to human disease and cancer.

## INTRODUCTION

The maintenance of genome integrity relies on accurate DNA duplication in all organisms. Any condition resulting in DNA replication perturbation gives rise to replication stress, which is a source of genetic instability, and a feature of pre-cancerous and cancerous cells (1,2). To deal with replication stress and protect arrested forks until replication resumes, eukaryotic cells have evolved a number of repair pathways collectively referred to as DNA damage response (DDR). One of the major natural impediments to the progression of replication forks is transcription (3–6). Encounters or conflicts between replication and transcription are unavoidable, as they compete for the same DNA template, so that collisions occur quite frequently (7). The main transcription-associated structures that can constitute a barrier to replication fork progression are R-loops (8). They are physiological structures consisting of an RNA–DNA hybrid and a displaced single-stranded DNA that, if deregulated or inaccurately removed, can cause a clash between the replisome and the RNA polymerase (4,9). Furthermore, whether deleterious R‐loops are formed or stabilized following replication-transcription collisions is currently under investigation (10). Although how precisely such replication-transcription collisions are managed is not completely understood, however, the fact that unscheduled R-loops severely distress the ongoing forks raised the possibility that some DNA replication associated factors can participate in preventing their accumulation or processing. Consistently with this hypothesis, it is emerging that defects in DNA repair factors, including BRCA1 and 2 (11–14), the Fanconi anaemia pathway (15,16), RECQ5 DNA helicase (17), Bloom syndrome helicase (18) and RNA/DNA helicase senataxin (19), or in the apical activator of the DDR, the ATM kinase (20), might directly or indirectly stabilize R-loops, potentially blocking replication fork progression (21).

Werner syndrome protein (WRN) is a well-known fork-protection factor that belongs to the RecQ family of DNA helicases (22–24). Mutations in the *WRN* gene cause the Werner syndrome (WS), a human disorder associated with chromosomal instability and cancer predisposition (25). WRN participates in several important DNA metabolic pathways, and plays its major function in genome stability maintenance, participating in the repair and recovery of stalled replication forks (26–29). A crucial player in the process that recognizes and stabilizes stalled forks is the ATR kinase, which phosphorylates a variety of proteins to trigger the replication checkpoint that coordinates accurate handling of perturbed replication forks (30). Several studies from our and other groups have envisaged a collaboration between WRN and the ATR pathway (31–34). Notably, WRN is phosphorylated in an ATR‐dependent manner upon replication stress (32,34,35); it is differently regulated by ATR and ATM to prevent double-strand breaks (DSBs) formation at stalled forks, and promote the failsafe recovery from replication arrest (32). Moreover, WRN helicase activity has been implicated in preserving integrity of common fragile sites (CFS) (36), which are the naturally occurring fork stalling sites (37). Therefore, these findings strongly support a role of WRN in facilitating replication fork progression of DNA regions affected by replication stress (38,39). Furthermore, our previous study showed that WRN plays a role as crucial regulator of the ATR-dependent checkpoint in response to mild form of replication stress (35). As WRN-deficient cells show impaired ATR-dependent CHK1 phosphorylation, stabilization of stalled forks is compromised leading to CFS instability (35). Although WRN, but not its helicase activity, is essential for establishing the replication checkpoint after short treatments with low-dose of aphidicolin (Aph), a selective inhibitor of replicative DNA polymerases, however, CHK1 activation is detected in WRN-deficient cells upon prolonged exposure to the drug (35), raising the possibility that a compensatory repair pathway is triggered. In line with this, it has been proposed that replication stress conditions that do not appear to induce DSBs, such as low-dose of Aph, elicits an ATM signaling in a way not completely understood (40).

Here, we report that WRN-deficient cells trigger an ATM signalling, which is responsible for CHK1 phosphorylation observed after prolonged Aph-induced replication perturbation. Moreover, we establish a key role of replication-transcription collisions and unscheduled R-loop accumulation in ATM pathway activation in WS cells. Finally, we demonstrate that, under conditions of mild replication stress, activation of ATM signalling is essential to limit R-loop-associated genomic instability in WRN-deficient cells.

## MATERIALS AND METHODS

### Cell cultures

AG11395 (WRN-deficient) human fibroblasts retrovirally-transduced with full length cDNA encoding wild-type WRN (WSWRN) or missense-mutant form of WRN with inactive helicase (WRN^K577M^) were generated as previously described (36). The SV40-transformed MRC5 fibroblast cell line (MRC5SV) was a generous gift from Patricia Kannouche (IGR, Villejuif, France). All the cell lines were maintained in Dulbecco's modified Eagle's medium (DMEM; Life Technologies) supplemented with 10% FBS (Boehringer Mannheim), and incubated at 37°C in a humidified 5% CO_2_ atmosphere.

### Immunofluorescence

Immunofluorescent detection of phospho-ATM was performed according to standard protocol with minor changes. Briefly, exponential growing cells were seeded onto Petri dishes, then treated (or mock-treated) as indicated, fixed in 3% formaldehyde/2% sucrose for 10 min, and permeabilized using 0.4% Triton X-100 for 10 min prior to incubation with 10% FBS for 1 h. After blocking, cells were incubated with the antibody against phospho-ATM-Ser1981 (Millipore) for 2 h at RT. Co-staining of phospho-ATM-Ser1981 and Cyclin A was performed fixing cells with 4% formaldehyde for 10 min, and permeabilized using 0.4% Triton X-100 for 10 min before being incubated with 10% FBS for 1 h. Then, cells were sequentially incubated with the following primary antibodies: phospho-ATM-Ser1981 (Millipore) and Cyclin A (Santa Cruz Biotechnology, Inc.) for 1 h at RT. Immunofluorescent detection of phospho-RPA was performed after pre-extraction with 0.4% Triton X-100, followed by fixation and blocking as indicated above. After blocking, cells were incubated with the antibody against phospho-RPA32-Ser33 (Bethyl laboratories) for 1 h at RT. Immunostaining for RNA–DNA hybrids was performed as described (10). Briefly, cells were fixed in 100% methanol for 10 min at –20°C, washed three times in PBS, pre-treated with 6 μg/ml of RNase A for 45 min at 37°C in 10 mM Tris–HCl pH 7.5 supplemented with 0.5 M NaCl, before blocking in 2% BSA/PBS overnight at 4°C. Cells were then incubated with the anti-DNA–RNA hybrid [S9.6] antibody (Kerafast) overnight at 4°C. After each primary antibody, cells were washed twice with PBS, and incubated with the specific secondary antibody: goat anti-mouse Alexa Fluor-488 or goat anti-rabbit Alexa Fluor-594 (Molecular Probes). The incubation with secondary antibodies were accomplished in a humidified chamber for 1 h at RT. DNA was counterstained with 0.5 μg/ml DAPI. Images were randomly acquired using Eclipse 80i Nikon Fluorescence Microscope, equipped with a VideoConfocal (ViCo) system. For each time point, at least 200 nuclei were acquired at 40× magnification. Phospho-ATM or S9.6 intensity per nucleus was calculated using ImageJ. Parallel samples incubated with either the appropriate normal serum or only with the secondary antibody confirmed that the observed fluorescence pattern was not attributable to artefacts.

### Dot blot analysis

Dot blot analysis was performed according to the protocol reported elsewhere (41). Genomic DNA was isolated by standard extraction with phenol/clorophorm/isoamylic alcohol (pH 8.0) followed by precipitation with 3 M NaOAc and 70% ethanol. Isolated gDNA was randomly fragmented overnight at 37°C with a cocktail of restriction enzymes (BsrgI, EcoRI, HindIII, XbaI) supplemented with 1 M Spermidin. After incubation, digested DNA was cleaned up with phenol/chloroform extraction and standard Ethanol precipitation. After sample quantification, 5 μg of digested DNA were incubated with RNase H overnight at 37°C as a negative control. Five micrograms of each sample were spotted onto a nitrocellulose membrane, blocked in 5% non-fat dry milk and incubated with the anti-DNA–RNA hybrid [S9.6] antibody (Kerafast) overnight at 4°C. Horseradish peroxidase-conjugated goat specie-specific secondary antibody (Santa Cruz Biotechnology, Inc.) was used. Quantification on scanned image of blot was performed using Image Lab software.

### DNA fiber analysis

Cells were pulse-labelled with 25 μM 5-chloro-2′-deoxyuridine (CldU) and 250 μM 5-iodo-2′-deoxyuridine (IdU) at specified times, with or without treatment as reported in the experimental schemes. Cells were or not pre-treated with the transcription inhibitor 5,6-dichloro-1-ß-d-ribofurosylbenzimidazole (DRB 50 μM for 1 h). DNA fibres were prepared and spread out as previously reported (35). For immunodetection of labelled tracks the following primary antibodies were used: anti-CldU (rat-monoclonal anti-BrdU/CldU; BU1/75 ICR1 Abcam) and anti-IdU (mouse-monoclonal anti-BrdU/IdU; clone b44 Becton Dickinson). The secondary antibodies were: goat anti-mouse Alexa Fluor 488 or goat anti-rat Alexa Fluor 594 (Molecular Probes). The incubation with antibodies was accomplished in a humidified chamber for 1 h at RT.

Images were acquired randomly from fields with untangled fibres using Eclipse 80i Nikon Fluorescence Microscope, equipped with a Video Confocal (ViCo) system. The length of labelled tracks were measured using the Image-Pro-Plus 6.0 software, and values were converted into kilobases using the conversion factor 1 μm = 2.59 kb as reported (35). A minimum of 100 individual fibres were analysed for each experiment and the mean of at least three independent experiments presented. Statistics were calculated using Graph Pad Prism Software.

### Statistical analysis

Statistical differences in all case were determined by two-tailed Student's *t* test. In all cases, not significant *P* > 0.05; *P < 0.05; **P < 0.01; ***P < 0.001; ****P < 0.0001.

## RESULTS

### WRN-deficient cells activates an ATM signalling in response to mild replication stress

**Figure 1.**
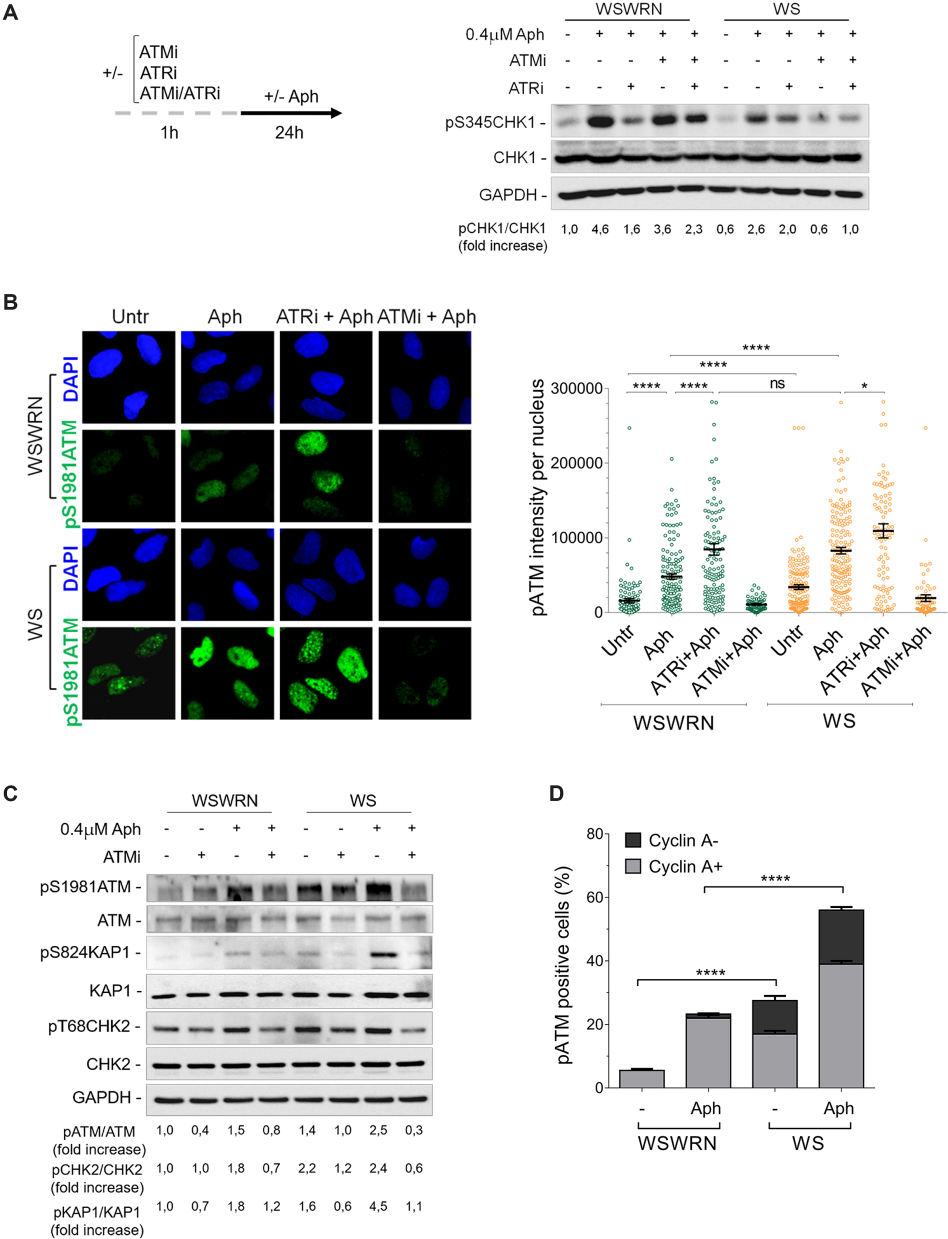
WRN deficiency leads to ATM pathway activation upon mild replication stress. (**A**) Western blot of CHK1 activation in total extracts of Werner syndrome (WS) and WS-corrected (WSWRN) cells treated as reported in the experimental design. The presence of activated, i.e. phosphorylated, CHK1 was assessed using S345 phospho-specific antibody (pS345). Total amount of CHK1 was determined with anti-CHK1 antibody. Equal loading was confirmed probing the membrane with anti-GAPDH antibody. The fold increase with respect to the wild-type (WSWRN) untreated of the normalized ratio of the phosphorylated CHK1/total CHK1 is reported for each cell line. Representative gel images of at least three replicates are shown. (**B**) Evaluation of ATM activation by immunofluorescence analysis in WSWRN and WS cells treated as in (A). The presence of activated, i.e. phosphorylated, ATM was assessed using S1981 phospho-specific antibody pATM (S1981). Nuclei were counterstained with DAPI. Representative images of cells stained for pATM are given. Dot plot shows pATM intensity per nucleus from three independent experiments. Horizontal black lines represent the mean ± SE. (ns, not significant; ****P < 0.0001; *P < 0.1; two-tailed Student's *t* test). (**C**) Western blot analysis of ATM, KAP1 and CHK2 activation in total extracts of WSWRN or WS cells exposed or not to ATMi 1 h prior to treatment with Aph. The presence of activated, i.e. phosphorylated, ATM, KAP1 or CHK2 was assessed using anti-pATM (S1981), anti-pKAP1 (S824) or anti-pCHK2 (T68) antibody. Total amount of ATM, KAP1 or CHK2 was determined with anti-ATM, anti-KAP1 or anti-CHK2 antibody. Anti-GAPDH antibody was used as loading control. The fold increase with respect to the wild-type (WSWRN) untreated of the normalised ratio of the phosphorylated ATM/total ATM, KAP1/total KAP1 or CHK2/total CHK2 is reported for each cell line. Representative gel images of at least three replicates are shown. (**D**) Analysis of pATM signal in WSWRN and WS cells treated or not with Aph. Cells were co-immunostained with antibodies against pATM (S1981) and Cyclin A. Bar graph shows the percentage of pATM positive cells in Cyclin A-positive (Cyclin A+) or negative (Cyclin A–) cells ± SE from three independent experiments. (****P < 0.0001; two-tailed Student's *t* test).

Loss of WRN results in replication checkpoint defects in response to short times of mild replication stress (35). However, prolonged exposure to low-dose of aphidicolin (Aph) activates CHK1 irrespective of the presence of WRN (35). Hence, we investigated whether ATM or ATR was responsible for CHK1 activation after prolonged treatment with Aph in WS cells. First, we examined CHK1 phosphorylation at Ser345 in an isogenic pair of uncorrected and WRN wild-type-corrected (WSWRN) SV40-transformed Werner syndrome (WS) fibroblasts (36), in which ATM (ATMi), ATR (ATRi) or both (ATMi/ATRi) were chemically inhibited. KU-55933 (42) and VE-821 (43) were used to selectively disrupt ATM or ATR kinase activity, respectively. Western blot analysis showed that Aph promoted CHK1 phosphorylation in both cell lines, although at reduced levels in WS cells (Figure 1A). Consistently with an earlier report (44), in WS cells complemented with wild-type WRN (WSWRN), CHK1 phosphorylation was not affected by inhibition of ATM after replication stress, but it was considerably lowered by ATRi (Figure 1A). However, and surprisingly, combined inhibition of ATM and ATR efficiently recovered CHK1 activation, indicating the involvement of other kinases (Figure 1A). By contrast, in WS cells treated with Aph, CHK1 activation is mainly ATM-dependent (Figure 1A). Essentially similar results were obtained using another selective inhibitor of the kinase activity of ATM, KU-60019 (Supplementary Figure S1) (45). Furthermore, as comparable amounts of Cyclin A, which marks S and G2 phases (46), were detected in WSWRN and WS cells, reducing levels of pCHK1 in WS cells are not attributable to a smaller S-phase population (Supplementary Figure S2).

This result prompted us to evaluate the autophosphorylation of ATM at Ser1981 (pATM), a widely accepted marker for ATM activation (47), in WRN-deficient cells. To this aim, WSWRN and WS cells were exposed to Aph, and ATRi or ATMi, then immunostained for pATM. Although Aph triggered ATM phosphorylation in both cell lines, fluorescence intensity of pATM was greater in WS cells than in WSWRN cells under unperturbed and treated conditions (Figure 1B). As expected, chemical inhibition of ATM lowered the pATM signal (Figure 1B). By contrast, ATRi enhanced pATM intensity in Aph-treated WSWRN cells, reaching values similar to those of WS cells (Figure 1B). Supporting the above observations, Western blot analysis showed the appearance of a band corresponding to pATM in both cell lines after treatment, which was reduced by KU-55933, but the level of Aph-induced pATM was higher in WS cells as compared to their corrected counterparts (Figure 1C).

Since immediately after its recruitment ATM kinase modifies multiple substrates (48,49) we examined the status of the downstream substrates, CHK2 and KAP1, by investigating their phosphorylation. In line with a previous report (40), we found that, in WSWRN cells, Aph caused CHK2 activation (pT68CHK2), which was modulated by ATM inhibition (Figure 1C). Notably, in WS cells, CHK2 phosphorylation was already evident under untreated conditions, but was not greatly increased by Aph (Figure 1C). However, CHK2 status was affected by KU-55933 with or without replication stress (Figure 1C). Interestingly, analysing phosphorylation of the more sensitive ATM-specific KAP1 at Ser824 site (50), we observed higher ATM-dependent activation of KAP1 after Aph treatment in WS cells respect to WSWRN cells (Figure 1C). This confirms that an ATM signalling is active.

To determine how rapidly the ATM activation occurred, we treated cells with Aph for different times. While in WSWRN cells ATM was only modestly phosphorylated at earlier time points, it was significantly activated at 16–24 h (Supplementary Figure S3A). Conversely, in WS cells, the levels of pATM intensity increased with time, reaching values higher than those observed at 16–24 h of Aph in WSWRN cells (Supplementary Figure S3A). In addition, there was no difference in cell cycle distribution between cell lines to justify the prompt accumulation of phosphorylated ATM in the absence of WRN (Supplementary Figure S3B). Furthermore, co-staining of pATM with Cyclin A indicated that ATM was activated mainly in Cyclin A-positive cells (Figure 1D and Supplementary Figure S4). Interestingly, a considerable portion of pATM signal was detected in Cyclin A-negative cells specifically in WS cell line with or without Aph treatment (Figure 1D and Supplementary Figure S4).

**Figure 2.**
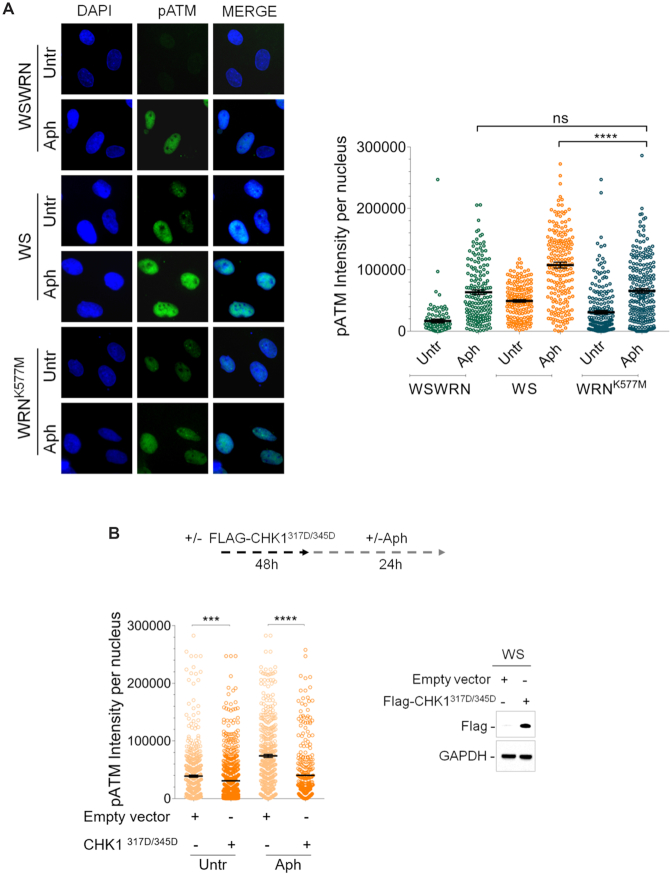
Evaluation of loss of WRN helicase activity or ectopic expression of pCHK1 on ATM activation. (**A**) Evaluation of ATM activation by immunofluorescence analysis in Werner syndrome (WS), WS-corrected (WSWRN) cells and WS cells expressing a mutant form of WRN affecting helicase function (WRN^K577M^) treated or not with Aph. Immunostaining was performed with an anti-pATM (S1981) antibody. Nuclei were counterstained with DAPI. Representative images are given. Dot plot shows pATM intensity per nucleus from three independent experiments. Horizontal black lines represent the mean ± SE. (ns, not significant; ****P < 0.0001; two-tailed Student's *t* test). (**B**) Evaluation of ATM activation by immunofluorescence analysis in WSWRN and WS cells transfected with an empty vector or a Flag-tagged CHK1^317/345D^, and treated or not with Aph 48 h post-transfection. Immunostaining was performed with an anti-pATM (S1981) antibody. Dot plot shows pATM intensity per nucleus from three independent experiments. Horizontal black lines represent the mean ± SE. (***P < 0.001; ****P < 0.0001; two-tailed Student's t test). Expression levels of Flag-CHK1^317/345D^ were determined by immunoblotting with anti-Flag antibody. Anti-GAPDH antibody was used to assess equal loading.

Since WRN helicase-defective cells phosphorylate early CHK1 upon mild replication stress (35), we asked whether, in this context, an ATM pathway was established. To this aim, WS fibroblasts or WS cells stably expressing the wild-type WRN (WSWRN) or WRN helicase-dead form (WRN^K577M^) (36) were treated or not with Aph, and subjected to immunostaining for pATM. We found that pATM signal intensity in WRN^K577M^ cells was similar to that observed in WS-corrected cells after treatment, but it was significantly lower respect to Aph-treated WS cells (Figure 2A). However, addition of UCN-01, a selective inhibitor of CHK1 (51), stimulated ATM phosphorylation both under unperturbed and treated conditions in WRN helicase-defective cells, but not in WSWRN cells (Supplementary Figure S5). This result suggests that lack of Werner helicase activity does not elicit an ATM signalling.

Next, to further prove that ATM signalling activation was related to loss of early CHK1 phosphorylation, we verified whether transfection with a construct expressing a mutant form of CHK1, in which Ser317 and Ser345 were changed into Asp (CHK1^317D/345D^) mimicking the phosphorylated status of the protein (52), could influence the ability of WS cells to activate ATM. As shown in Figure 2B, transfection of CHK1^317D/345D^ protein significantly abrogated ATM phosphorylation in WS cells both under unperturbed and Aph-treated conditions (Figure 2B). This demonstrates that ectopic expression of phospho-mimic CHK1 compensates for defective ATR pathway in WRN-deficient cells, thus reducing the need to establish an ATM signalling. Consistently with this, expression of the CHK1 mutant decreased the levels of CHK2 phosphorylation in WS cells (Supplementary Figure S6).

These results provide evidence that an ATM signalling is hyper-activated in WRN-deficient cells, leading to CHK1 phosphorylation after mild replication stress.

### Inhibition of ATM kinase activity results in enhanced chromosomal damage and cell death in WRN-deficient cells after mild replication stress

**Figure 3.**
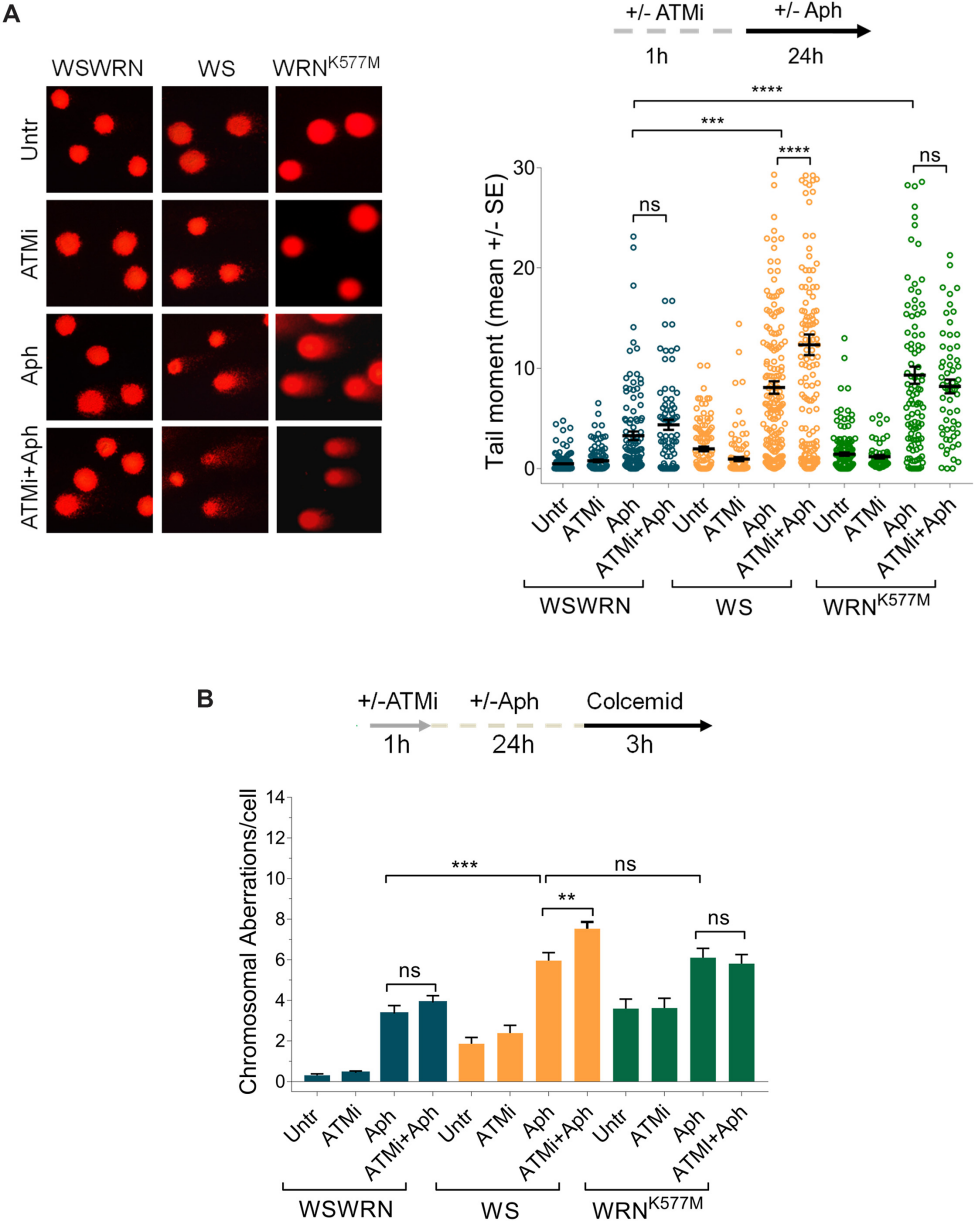
Inhibition of ATM enhances DNA damage and chromosomal aberrations in WRN-deficient cells upon mild replication perturbation. (**A**) Analysis of DNA damage accumulation evaluated by alkaline Comet assay. Werner syndrome (WS), WS-corrected (WSWRN) cells and WRN helicase-defective (WRN^K577M^) cells were treated as reported in the scheme, and then subjected to Comet assay. Representative images are given. Graph shows data presented as mean tail moment ± SE from three independent experiments. Horizontal black lines represent the mean. (ns, not significant; ***P < 0.001; ****P < 0.0001; two-tailed Student's *t* test). (**B**) Analysis of chromosomal aberrations in WSWRN, WS and WRN^K577M^ cells treated as shown in the experimental scheme. Metaphases were collected by colcemid. Next, cells were fixed and processed as reported in ‘Supplementary Materials and Methods’. Bar graph shows the number of chromosomal aberrations per cell ± SE from three independent experiments. (ns, not significant; **P < 0.01; ***P < 0.001; two-tailed Student's *t* test).

Once demonstrated that mild replication stress stimulates an ATM signalling in WRN-deficient cells, we investigated the consequences of its disruption on genome stability. We first examined the effect of ATM inhibition on DNA damage accumulation performing the alkaline Comet assay in WSWRN, WS and WRN^K577M^ cells. As expected, spontaneous DNA damage sligthly increased in WS and WRN^K577M^ cells respect to WSWRN cell line (Figure 3A). Moreover, after treatment, WS and WRN^K577M^ cells exhibited markedly enhanced comet tail moment, reaching values significantly higher than those of WSWRN cells (Figure 3A). Interestingly, addition of KU-55933 to the Aph-treated WS cells heightned the levels of DNA damage respect to the treatment alone (Figure 3A). This finding was confirmed using the ATM kinase inhibitor KU-60019 (Supplementary Figure S7). However, and in agreement with our previous data (53), Aph did not induce appreciable amount of double-strand breaks (DSBs), as assessed by neutral Comet assay (Supplementary Figure S6). Conversely, inhibition of ATM activity significantly increased DSB formation in the absence of WRN after mild replication stress (Supplementary Figure S8).

Taking into account the characteristic chromosomal instability of WRN-deficient cells after mild replication stress (36), we examined the effect of ATM inhibition on chromosomal aberrations. As expected, WS and WRN^K577M^ cells exhibited higher levels of spontaneous chromosomal aberrations respect to WSWRN cells, which were not influenced by ATM inhibition (Figure 3B and Supplementary Figure S9). Moreover, Aph treatment increased chromosomal damage in WS and WRN^K577M^ cells more than in WSWRN cells (Figure 3B and Supplementary Figure S9). However, and importantly, while ATM inhibition enhanced chromosomal aberrations in WRN knockout cell line, in WRN^K577M^ cells did not (Figure 3B and Supplementary Figure S9). Overall, these findings indicate that chromosomal instability is exacerbated by abrogation of ATM pathway only in WRN-deficient cells.

As an additional sign of chromosomal damage, we examined the presence of micronuclei. As shown in Supplementary Figure S10A, an elevated percentage of cells with micronuclei was found in WS and WRN^K577M^ cell lines. However, and interestingly, inhibition of ATM increased the number of cells with micronuclei only in Aph-treated WS cells (Supplementary Figure S10A). Finally, we evaluated the effect of ATM inhibition on cell viability. The fluorescence-based LIVE/DEAD assay showed that loss of WRN or WRN helicase activity led to marked cell death, enhanced by KU-55933 only in WS cells (Supplementary Figure S10B). Furthermore, although Aph treatment compromised cell viability in all cell lines, however, this effect was exacerbated by ATM inhibition exclusively in the absence of WRN (Supplementary Figure S10B).

Altogether, these experiments demonstrate that WRN-deficient cells exhibit a high sensitivity to ATM activity inhibition, especially after Aph-induced replication stress, further enhancing genomic instability.

### R-loop accumulation is responsible for ATM signalling activation in WRN-deficient cells

**Figure 4.**
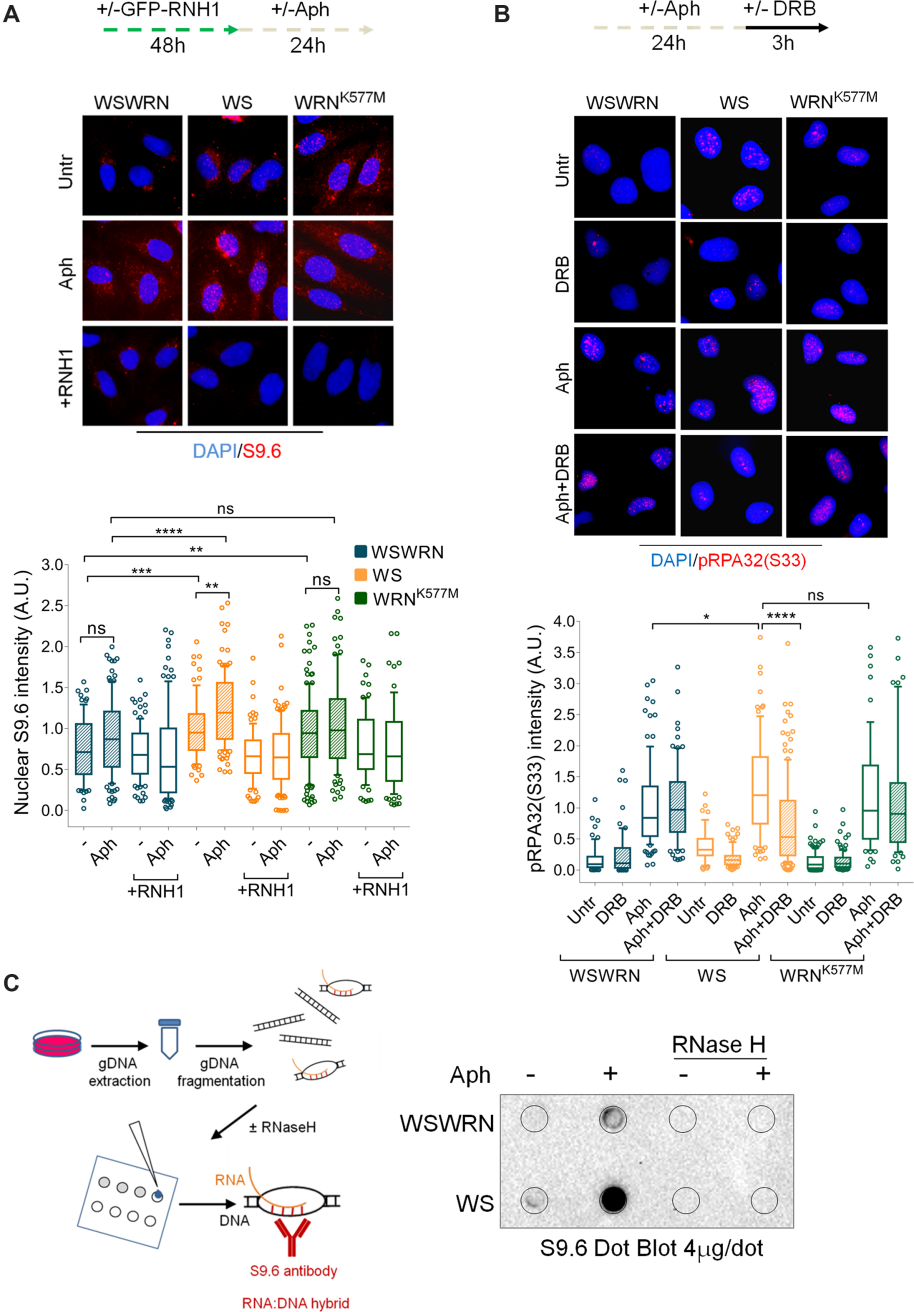
Loss of WRN results in R-loop accumulation. (**A**) Evaluation of R-loop accumulation by immunofluorescence analysis. Werner syndrome (WS), WS-corrected (WSWRN) cells and WRN helicase-defective (WRN^K577M^) cells were treated as reported in the experimental design. Next, cells were fixed and stained with anti-RNA–DNA hybrid S9.6 monoclonal antibody. Nuclei were counterstained with DAPI. Representative images are given. Box plot shows nuclear S9.6 fluorescence intensity. Box and whiskers represent 25–75 and 10–90 percentiles, respectively. The line represents the median value. Data are presented as means of three independent experiments. Horizontal black lines represent the mean. Error bars represent standard error. (ns, not significant; **P < 0.01; ***, *P* < 0.001; ****P < 0.0001; two-tailed Student's *t* test). (**B**) Evaluation of RPA32 phosphorylation on Ser33 by immunofluorescence analysis. WSWRN, WS and WRN^K577M^ cells were treated as reported in the experimental scheme. After pre-extraction, cells were fixed and subjected to immunostaining with Ser33 phospho-specific RPA32 (pS33RPA) antibody. Nuclei were counterstained with DAPI. Representative images are given. Box plot shows nuclear pRPA32 (S33) fluorescence intensity per nucleus. Box and whiskers represent 25–75 and 10–90 percentiles, respectively. The line represents the median value. Data are presented as means of three independent experiments. Horizontal black lines represent the mean. Error bars represent standard error. (ns, not significant; *P < 0.1; ****P < 0.0001; two-tailed Student's *t* test). (**C**) Dot blotting to confirm R-loop accumulation. Genomic DNA isolated from WSWRN and WS cells, treated as reported in the experimental design and processed as described in ‘Materials and Methods’, was spotted onto nitrocellulose membrane. The membrane was probed with anti-RNA–DNA hybrid S9.6 monoclonal antibody. Treatment with RNase H was used as a negative control. Representative gel images of at least three replicates are shown.

Several studies have shown that a main source of genome instability comes from collisions between replication and transcription due to excessive formation of DNA–RNA hybrids (7). Furthermore, an R-loop-dependent ATM activation by transcription-blocking lesions was reported in the DNA damage response (10,20). Hence, our results prompted us to evaluate direct R-loop accumulation in the cells by immunofluorescence using the anti-RNA–DNA hybrid S9.6 monoclonal antibody (10,54). Our analysis showed that spontaneous levels of S9.6 fluorescence intensity in both WS and WRN^K577M^ cells were significantly higher than those in WSWRN cells (Figure 4A). The phenotype of WS cells agrees with that obtained in HeLa cells in which WRN was depleted by RNA interference (18). However, while no enrichment of the S9.6 nuclear signal was observed after mild replication stress in WRN^K577M^ cells respect to WSWRN cells, it significantly increased in WS cells (Figure 4A). Notably, overexpression of ectopic GFP-RNaseH1, a ribonuclease that specifically degrades the RNA moiety of RNA–DNA hybrids in the nucleus (55), strongly suppressed the S9.6 staining, which in WS cells was normalized to that of their corrected counterparts, confirming the R-loop accumulation in Aph-treated WRN-deficient cells (Figure 4A). To further strengthen this observation, we stained cells with a phosphorylated form of RPA32 (pS33RPA32), which is an established marker of stalled replication forks and sites of DNA damage, recently correlated with R-loop formation (55). Immunofluorescence analysis showed that the nuclear intensity of pRPA was significantly enriched in Aph-treated WS cells respect to WSWRN and WRN^K577M^ cells (Figure 4B). As expected, and basing on the acquired flow cytometry data, inhibition of transcription by 5,6-dichloro-1-ß-d-ribofurosylbenzimidazole (DRB), an inhibitor of RNA chain elongation, at a concentration that inhibits transcription but does not greatly perturb cell cycle progression ((56) and Suppl Figure 11), significantly reduced pRPA nuclear intensity in the absence of WRN (Figure 4B). Hence, we concluded that, under our conditions, a substantial fraction of phospho-RPA signal correlates with R-loop formation in WS cells. To support the notion that loss of WRN increased R-loop accumulation within nuclear DNA, we isolated genomic DNA from WSWRN and WS cells, and performed dot blot analysis. Consistent with our fluorescence results, we found that the amount of S9.6 signal was higher in WS cells as compared to their corrected counterparts (Figure 4C). Importantly, the S9.6 signal was strongly diminished after RNaseH treatment (Figure 4C) (41).

**Figure 5.**
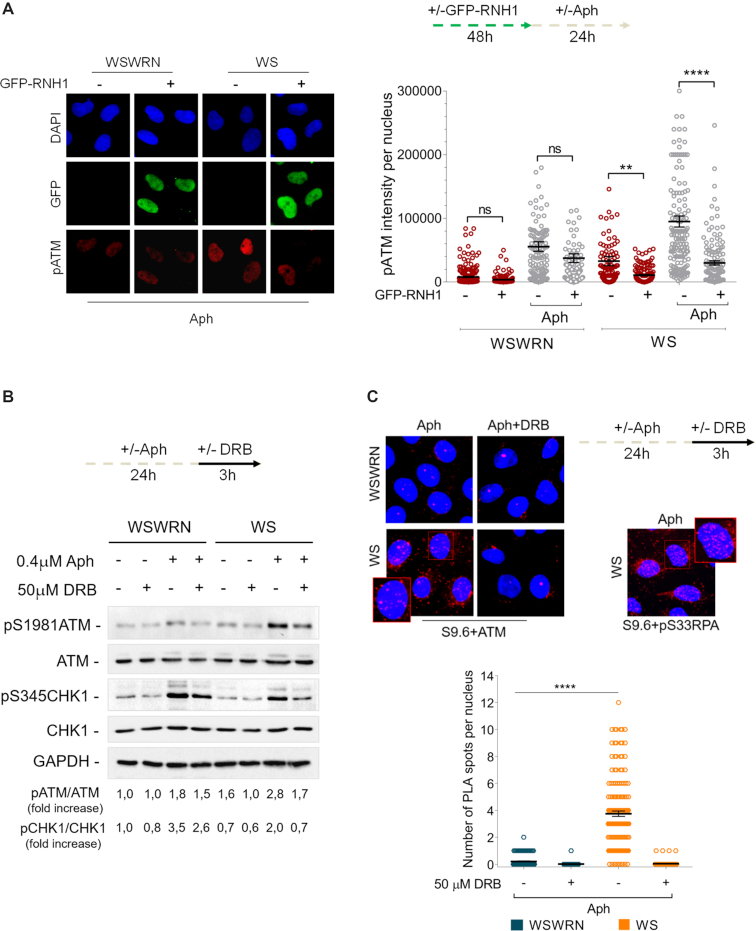
Analysis of R-loop accumulation or transcription inhibition and ATM activation. (**A**) Evaluation of ATM activation by immunofluorescence analysis in Werner syndrome (WS) and WS-corrected (WSWRN) cells treated as reported in the experimental design after transfection with GFP-tagged RNaseH1. Immunostaining was performed with anti-pATM (S1981) antibody. Nuclei were counterstained with DAPI. Representative images are given. Dot plot shows pATM intensity per nucleus from three independent experiments. Horizontal black lines represent the mean ± SE. (ns, not significant; **P < 0.01; ****P < 0.0001; two-tailed Student's *t* test). **(B)** Western blot detection of ATM and CHK1 activation in total extracts of Werner syndrome (WS) and WS corrected (WSWRN) cells treated as described in the experimental design. The presence of activated, i.e. phosphorylated, ATM, or CHK1 was assessed using anti-pATM (S1981), or anti-pCHK1 (S345) antibody. Total amount of ATM and CHK1 were determined with anti-ATM or anti-CHK1 antibody. Anti-GAPDH antibody was used as loading control. The fold increase with respect to the wild-type (WSWRN) untreated of the normalised ratio of the phosphorylated ATM/total ATM or CHK1/total CHK1 is reported for each cell line. Representative gel images of at least three replicates are shown. (**C**) Detection of physical interaction between R-loops and ATM by a fluorescence based assay. Werner syndrome (WS) and WS corrected (WSWRN) cells were treated as indicated in the experimental design, and then subjected to PLA assay as described in ‘Supplementary Materials and Methods’. Cells were stained with anti-RNA–DNA hybrid S9.6 antibody, and an antibody raised against ATM. Each red spot represents a single interaction between R-loops (S9.6) and ATM. No spot has been revealed in cells stained with each single antibody (negative control). Interaction between pRPA32 (pS33RPA) and R-loops detected by PLA was used as positive control. Nuclei were counterstained with DAPI. Representative images are given. Insets show enlarged nuclei for a better evaluation of PLA spots. Dot plot shows the number of PLA spots per nucleus. Horizontal black lines represent the mean ± SE. (****P < 0.0001; two-tailed Student's *t* test).

Hence, the next question was whether ATM signalling hyper-activation was correlated to the accumulation of R-loops in WRN-deficient cells. We therefore examined the effect of RNaseH1 overexpression in WSWRN and WS cells, treated or not with Aph, on the levels of pATM fluorescence intensity. As can be seen in Figure 5A, the intensity of pATM signal induced by loss of WRN, both in unperturbed and replication stress conditions, was strongly reduced after RNaseH1 plasmid transfection, but no effect was observed in WSWRN cells. Similar results were obtained treating the cells with DRB (Supplementary Figure S12). Consistently, Western blot analysis revealed that transcription inhibition greatly decreased the band intensity of pATM in Aph-treated WS cells (Figure 5B). Once demonstrated that ATM activation is mediated by R-loops, we investigated the impact of transcription inhibition on ATM-mediated CHK1 phosphorylation. As expected, upon Aph treatment, a clear activation of CHK1 was observed in both cell lines (Figure 5B). However, the levels of pCHK1 drastically dropped in WRN-deficient cells when Aph was incubated with DRB (Figure 5B). This confirms that ATM-mediated CHK1 is activated in an R-loop-dependent manner upon mild replication stress in WS cells.

The above observation raised an interesting possibility that R-loops could contribute to the retention/recruitment of ATM in chromatin. Hence, we used the in situ proximity ligation assay (PLA), a fluorescence method that allows to detect physical interactions (57), to explore the co-localization of ATM at/near R-loops. WSWRN and WS cells were incubated with Aph and DRB, then subjected to PLA using anti-ATM and anti-DNA–RNA (S9.6) antibodies. Our analysis showed increased number of PLA dots following Aph treatment, which were almost completely suppressed by DRB treatment (Figure 5C), indicating spatial proximity between R-loops and ATM in chromatin.

Moreover, taking into account that pATM and pRPA form nuclear foci, and that pRPA interacts with R-loops (55), we assessed the pRPA/pATM co-localization to demonstrate the presence of the phosphorylated form of ATM at R-loops. Immunofluorescence analysis showed that the majority of the pATM-positive cells co-localized with pRPA in WS cells in unperturbed conditions and in response to Aph-induced mild replication stress (Supplementary Figure S13). This result strongly reinforces the possibility that under our conditions ATM signalling activation is R-loop-dependent.

### ATM signalling activation depends on XPG-dependent processing of R-loops in WRN-deficient cells

**Figure 6.**
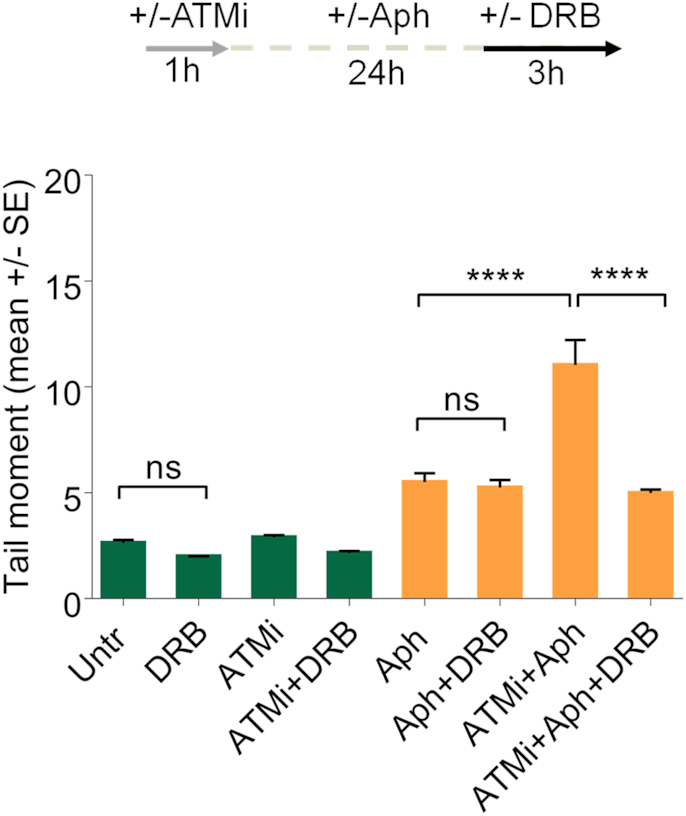
R-loop-dependent ATM activation in WS cells after mild replication stress. Analysis of DSB accumulation evaluated by neutral Comet assay in WS cells subjected to chemical inhibition of ATM and transcription as reported in the experimental scheme. After treatment, cells were harvested and subjected to neutral Comet assay. Dot plot shows data presented as mean tail moment ± SE from three independent experiments. Horizontal black lines represent the mean. (ns, not significant; ****P < 0.0001; two-tailed Student's *t* test).

ATM activation has primarily been viewed as a response to DSBs (58). Although, ATM phosphorylation is not associated with DNA breaks in WS cells, however, loss of ATM activity induced a significant amount of DSBs after mild replication stress (Supplementary Figure S8). Hence, we asked whether DNA breaks could depend on replication-transcription conflicts. To address this point, we evaluated DSB formation in WRN-deficient cells exposed to DRB after ATM inhibition. The neutral Comet assay showed that DRB significantly reduced the levels of DSBs in Aph-treated WS cells (Figure 6), suggesting that DNA breaks are largely R-loop-dependent.

**Figure 7.**
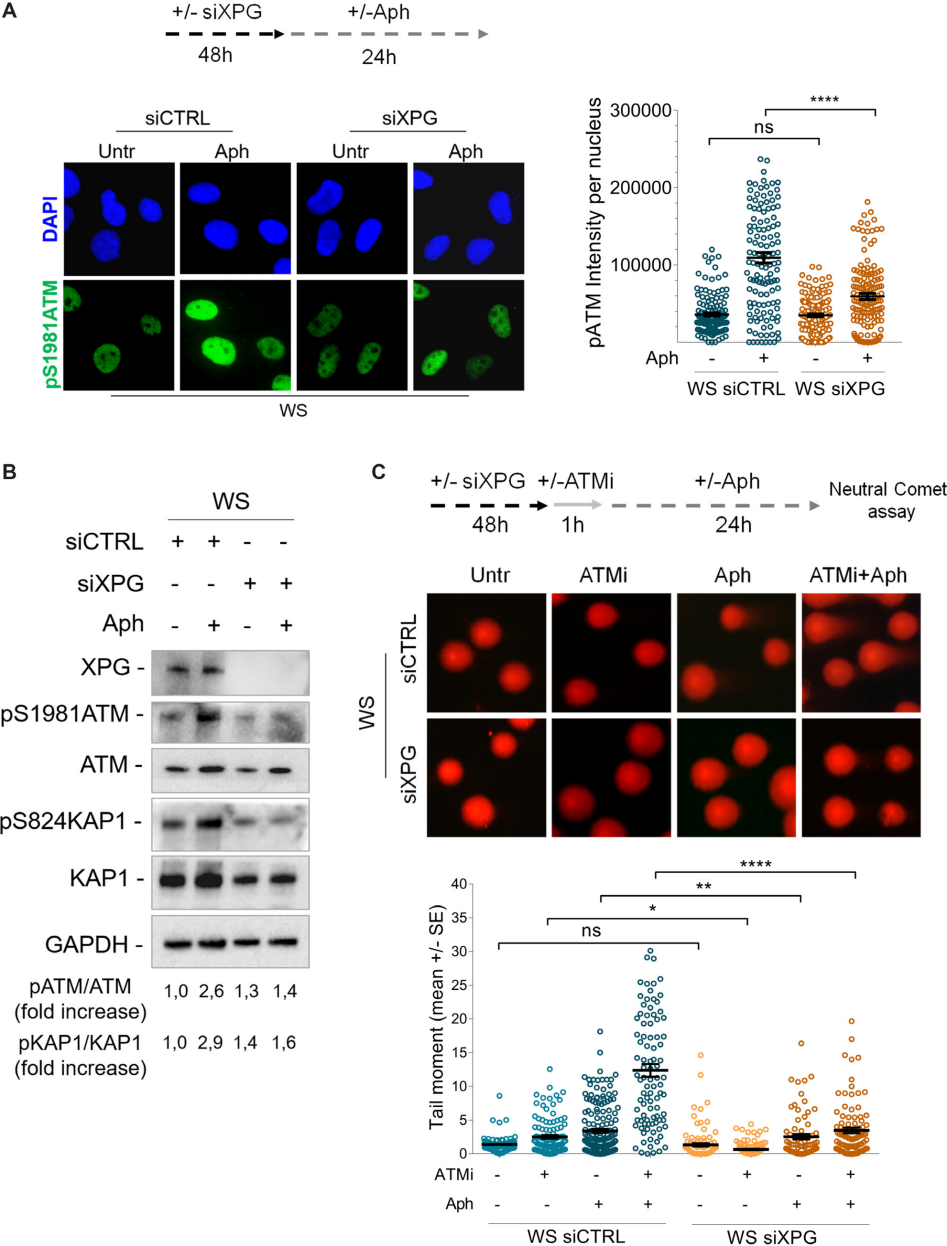
XPG-dependent processing of R-loops leads to ATM activation in WS cells. (**A**) Evaluation of ATM activation upon XPG depletion by immunofluorescence. Werner syndrome (WS) cells were transfected with control siRNAs (siCTRL) or siRNAs directed against XPG (siXPG). After 48 hours cells were treated as reported in the experimental scheme. Immunostaining was performed with an anti-pATM (S1981) antibody. Representative images are given. Dot plot shows pATM intensity per nucleus from three independent experiments. Horizontal black lines represent the mean ± SE. (ns, not significant; ****P < 0.0001; two-tailed Student's *t* test). (**B**) Western blot analysis of ATM, KAP1 activation in total extracts of WS cells depleted of XPG and treated as reported in the experimental design. Expression levels of XPG were determined by immunoblotting with anti-XPG antibody. The presence of activated, i.e. phosphorylated, ATM or KAP1 was assessed using anti-pATM (S1981) or anti-pKAP1 (S824) antibody. Total amount of ATM or KAP1 was determined with anti-ATM or anti-KAP1 antibody. Anti-GAPDH antibody was used as loading control. The fold increase with respect to the WS untreated of the normalised ratio of the phosphorylated ATM/total ATM or KAP1/total KAP1 is reported for each cell line. Representative gel images of at least three replicates are shown. (**C**) Analysis of DSB accumulation by neutral Comet assay. WS cells depleted of XPG and in which ATM is inhibited were treated as reported by the experimental scheme, and then subjected to neutral Comet assay. Representative images are given. Dot plot shows data presented as mean tail moment ± SE from three independent experiments. Horizontal black lines represent the mean. (ns, not significant; *P < 0.1; **P < 0.01; ****P < 0.0001; two-tailed Student's *t* test).

A previous work demonstrated that R-loops are actively processed into DSBs by the endonucleases XPG and XPF (59). Thus, we tested the hypothesis that ATM activation could depend on the presence of transient DSBs formed by XPG on R-loops that are rapidly repaired when ATM is active. To verify this, first, we examined pATM signal intensity by indirect immunofluorescence in WS cells, treated or not with Aph, in which XPG has been depleted by RNAi. Our analysis showed that XPG knockdown robustly reduced the levels of pATM intensity in WRN-deficient cells upon mild replication stress (Figure 7A), suggesting that loss of cleavage by XPG affects ATM activation. Consistent with reduced ATM activation, we observed a decrease in KAP1 phosphorylation after Aph-treatment in XPG-depleted WS cells (Figure 7B). Next, to further support the idea that XPG may be able to process R-loops producing transient DSBs, we performed a neutral Comet assay in WS cells in which both XPG and ATM activities are abolished. Interestingly, we found a significant suppression of neutral comet tail moment in XPG-depleted WS cells, providing a direct evidence for XPG-dependent DSB formation (Figure 7C).

Altogether, these data are consistent with the possibility that transient DSBs resulting from processing of R-loops by XPG leads to ATM signalling activation in WS cells.

### Impairment of replication fork progression in WRN-deficient cells is alleviated by transcription inhibition

**Figure 8.**
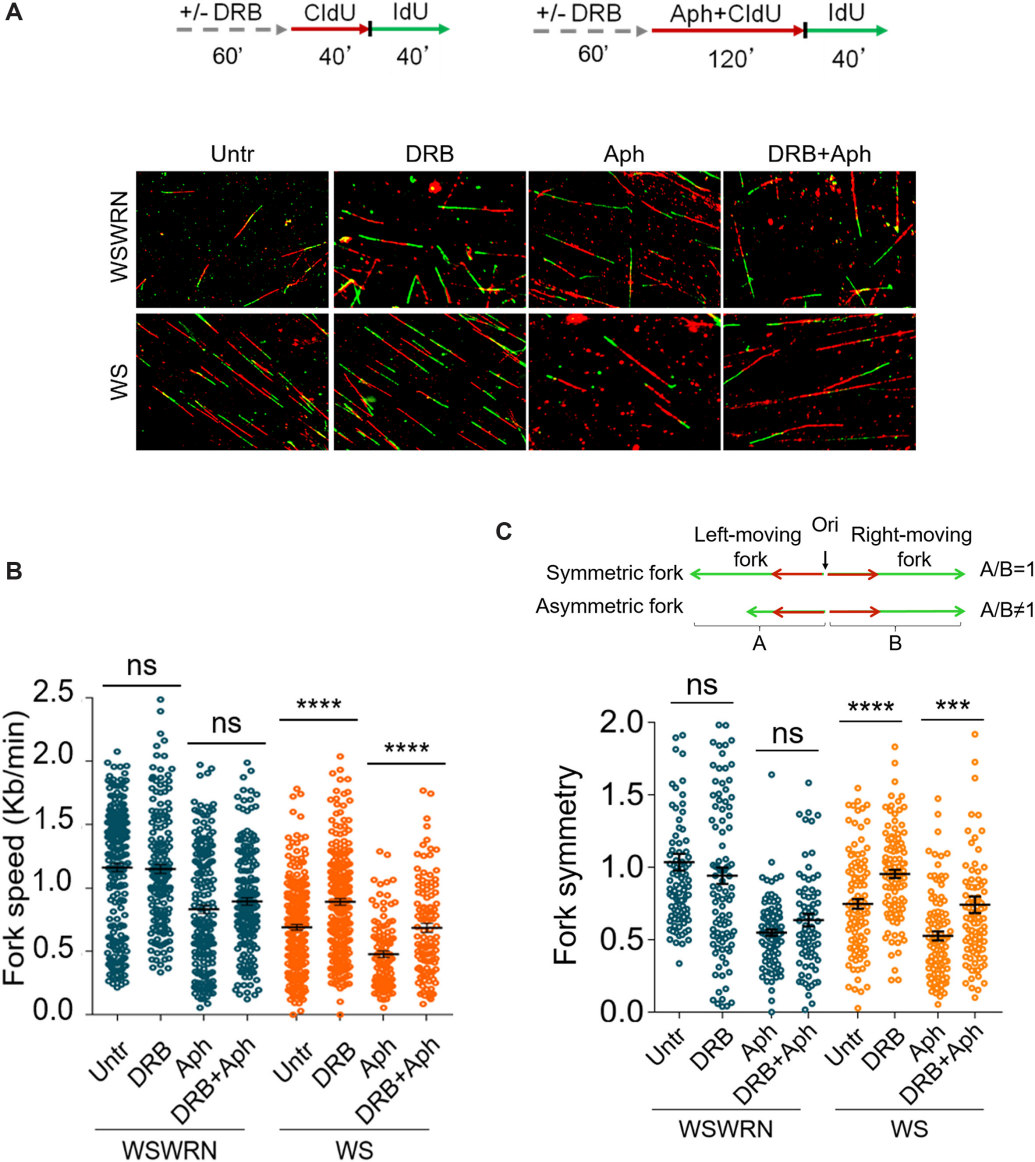
Transcription inhibition alleviates replication fork progression defect in WRN-deficient cells. (**A**) Experimental scheme of dual labelling of DNA fibers in Werner syndrome (WS) and WS-corrected (WSWRN) cells. Cells were pre-treated with DRB, then pulse-labelled with CldU and treated or not with Aph. After washing, cells were pulse-labelled with IdU as indicated. Representative DNA fiber images are shown. (**B**) Graph shows the analysis of replication fork velocity (fork speed) in the cells. The length of the green tracks were measured. Mean values are represented as horizontal black lines. (ns, not significant; ****P < 0.0001; two-tailed Student's *t* test). (**C**) Graph shows the evaluation of fork symmetry in the cells as reported in the scheme. Mean values are represented as horizontal black lines. (ns, not significant; ***P < 0.001; ****P < 0.0001; two-tailed Student's *t* test).

It is still unclear whether formation of R-loops is the cause or the consequence of collisions of replication forks with transcribing DNA (10,60). Hence, to substantiate our observation that ATM activation depends on R-loop-mediated replication-transcription conflicts, we investigated whether preventing transcription-associated structure accumulation alleviated replication fork progression perturbation in WRN-deficient cells, under conditions of prolonged mild replication stress. To this purpose, we used DNA fiber assay and examined replication fork dynamics at single-molecule resolution to evaluate the rate and symmetry of fork progression in Aph-treated WSWRN and WS cells, in which transcription was inhibited by DRB. We sequentially labelled cells with the thymidine analogues 5-chloro-2′-deoxyuridine (CldU) and 5-iodo-2′-deoxyuridine (IdU) as described in the experimental scheme (Figure 8A). Under normal growth conditions, and in agreement with previous data (35), loss of WRN led to a reduction of fork speed respect to WSWRN cells (Figure 8B). However, upon long-term exposure to Aph, fork velocity similarly decreased in both cell lines (Figure 8B). Interestingly, preventing R-loop accumulation, pre-treating cells with DRB, resulted in a significant enhancement in the fork progression rate only in WS cells (Figure 8B). Consistently, fork symmetry was significantly ameliorated after transcription inhibition only in WRN-deficient cells (Figure 8C).

These data imply that, under prolonged mild replication stress, loss of WRN impairs replication fork progression, but counteracting transcription-associated structure formation alleviates this defect.

### Loss of WRN leads to transcription- and R-loop-dependent genomic instability

**Figure 9.**
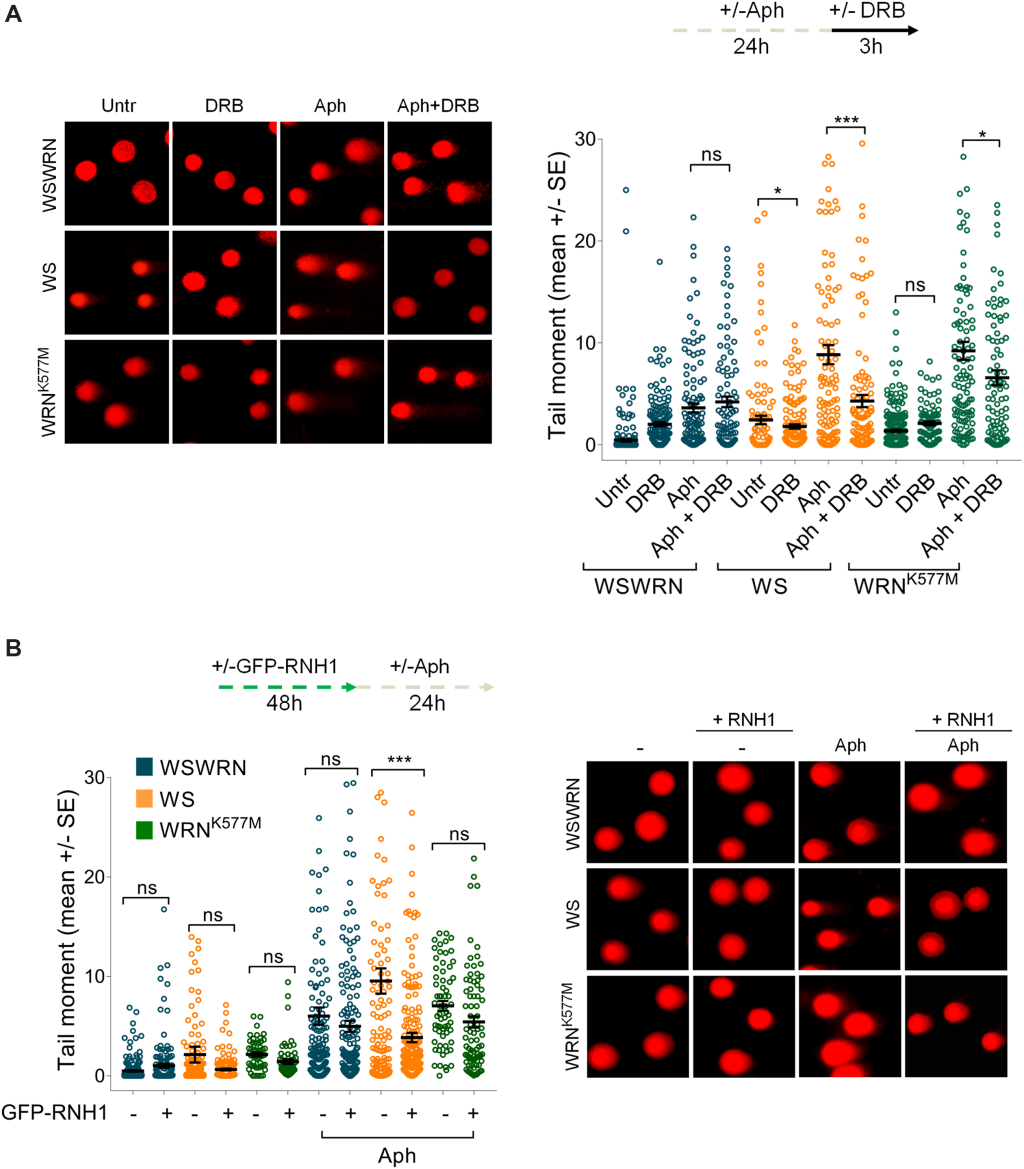
Loss of WRN leads to transcription and R-loop-associated DNA damage accumulation upon mild replication stress. (**A**) Analysis of the effect of transcription inhibition on DNA damage accumulation evaluated by alkaline Comet assay. Werner syndrome (WS), WS-corrected (WSWRN) cells and WRN helicase-defective (WRN^K577M^) cells were treated as reported in the experimental scheme, and subjected to Comet assay. Representative images are given. Graph shows data presented as mean tail moment ± SE from three independent experiments. Horizontal black lines represent the mean. (ns, not significant; *P < 0.1; ***P < 0.001; two-tailed Student's *t* test). (**B**) Evaluation of the effect of transfection of a vector expressing GFP-tagged RNaseH1 on DNA breakage. WSWRN, WS and WRN^K577M^ cells were treated as described in the experimental scheme, and subjected to alkaline Comet assay. Representative images are given. Graph shows data presented as mean tail moment ± SE from three independent experiments. Horizontal black lines represent the mean. (ns, not significant; ***P < 0.001; two-tailed Student's *t* test).

As replication-transcription conflicts play a key role in promoting R-loop-mediated genomic instability (7), and given that DRB prevents R-loop formation, we wondered whether DNA damage accumulation observed in WRN-deficient cells (Figure 3A) was dependent on transcription and RNA–DNA hybrids. To this aim, we performed an alkaline Comet assay in WSWRN, WS and WRN^K577M^ cells incubated with Aph and/or DRB. As expected, DRB did not affect the amount of DNA damage in WSWRN cells, and neither in WRN^K577M^ cells after mild replication stress (Figure 9A). By contrast, DRB significantly suppressed the levels of DNA damage in WS cells under unperturbed and Aph-treated conditions (Figure 9A). Similar results were obtained using cordycepin, confirming that the effect is not specific of the inhibitor rather than mediated by transcription (our unpublished observations). Importantly, overexpression of RNaseH1 significantly attenuated DNA damage only in WS cells after Aph-treatment, as evidenced from alkaline Comet assay (Figure 9B and Supplementary Figure S14).

**Figure 10.**
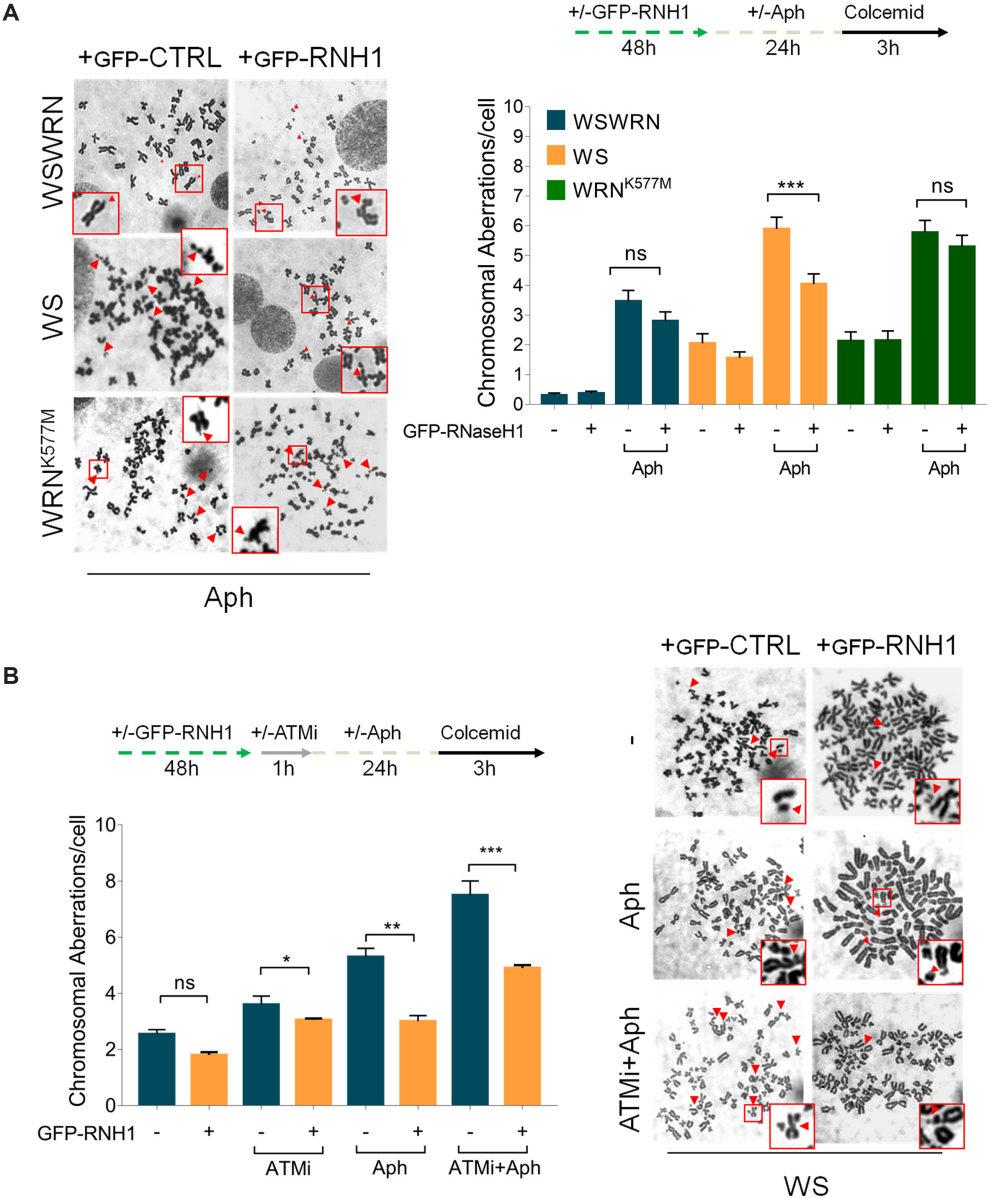
GFP-RNaseH1 overexpression suppresses chromosomal damage upon ATM inhibition in Aph-treated WS cells. (**A**) Analysis of chromosomal aberrations in Werner syndrome (WS), WS-corrected (WSWRN) cells and WRN helicase-defective (WRN^K577M^) cells treated as reported in the experimental scheme after transfection of a vector expressing GFP-tagged RNaseH1. Metaphases were collected by colcemid. Next, cells were fixed and processed as reported in ‘Supplementary Materials and Methods’. Representative images are given. Red arrows indicate chromosomal aberrations. Insets show enlarged metaphases for a better evaluation of chromosomal aberrations. Bar graph shows the number of chromosomal aberrations per cell. Data are presented as means of three independent experiments. Horizontal black lines represent the mean ± SE. (ns, not significant; ***P < 0.001; two-tailed Student's *t* test). (**B**) Analysis of chromosomal aberrations upon GFP-RNaseH1 overexpression and ATM inhibition in WS cells. The experimental set-up is shown. Metaphases were collected by colcemid. Next, cells were fixed and processed as described in ‘Supplementary Materials and Methods’. Representative images are given. Red arrows indicate chromosomal aberrations. Insets show enlarged metaphases for a better evaluation of chromosomal aberrations. Bar graph shows the number of chromosomal aberrations per cell. Data are presented as means of three independent experiments. Horizontal black lines represent the mean ± SE. Error bars represent standard error. (ns, not significant; **P < 0.01; two-tailed Student's *t* test).

Since loss of WRN causes sensitivity to mild replication stress (35,36) and Figure 3B, and given that WS cells accumulate R-loops after Aph treatment (Figure 4A), we asked whether chromosomal damage could depend on RNA–DNA hybrids. To test this possibility, we examined the effect of RNaseH1 overexpression on chromosomal aberrations in WSWRN, WS and WRN^K577M^ cells. We found that, after RNaseH1 plasmid transfection, only in WS cells the frequency of chromosomal aberrations was significantly reduced with respect to WRN^K577M^ and WSWRN cells upon mild replication stress (Figure 10A), confirming that R-loops can contribute to genomic instability in WRN-deficient cells.

**Figure 11.**
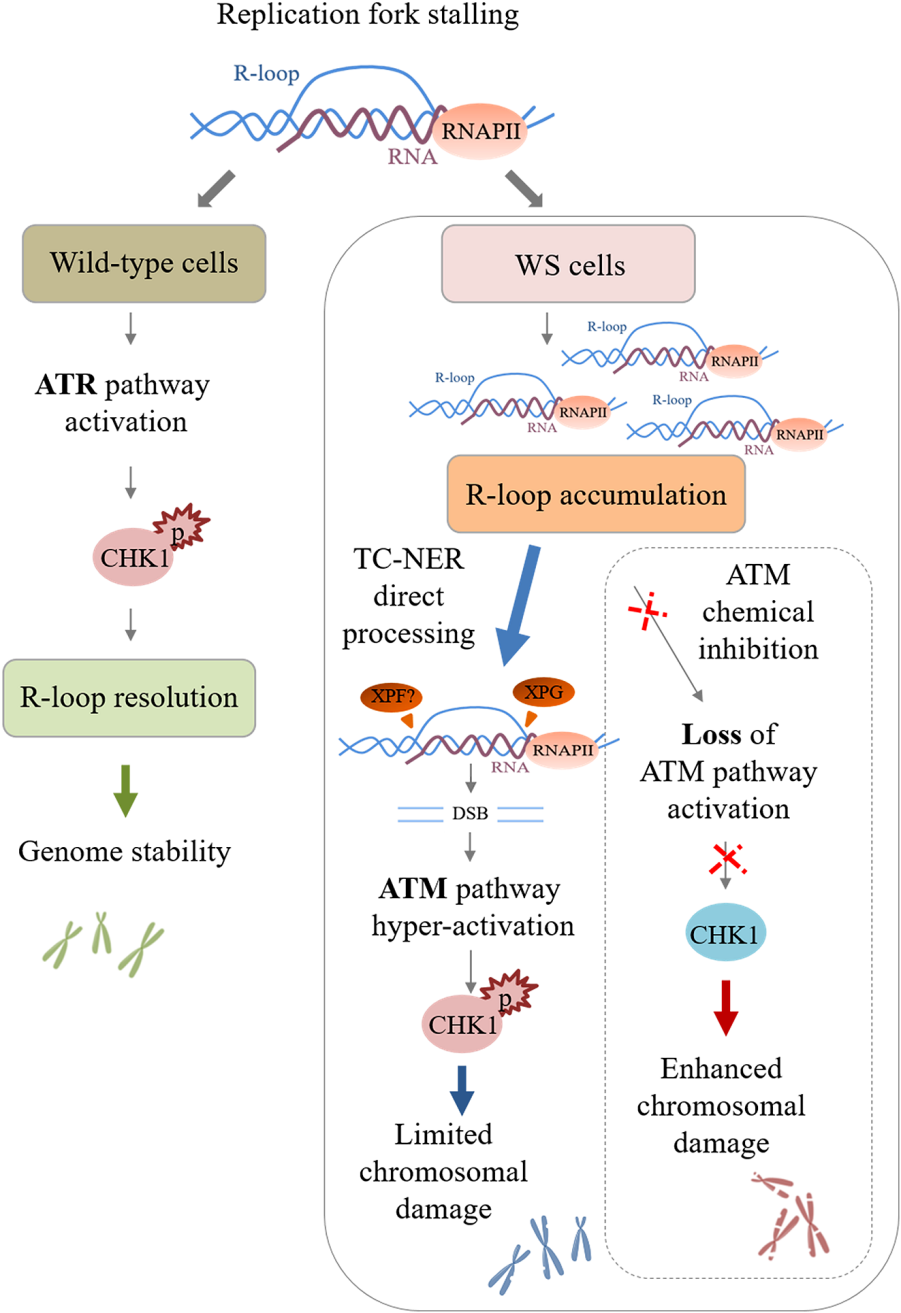
Model for a role of ATM pathway in limiting genome instability in WS cells. After replication fork stalling, the presence of WRN leads to a correct ATR-dependent CHK1 activation that ensures R-loop resolution and thus genome stability. In the absence of WRN, although an aberrant accumulation of R-loops is detected, a direct processing of these structures by the endonuclease XPG induces transient DSB formation, leading to ATM pathway hyper-activation. Under these conditions, active ATM mediates CHK1 phosphorylation that is crucial to limit chromosomal damage in WS cells. However, when ATM kinase is chemically inhibited, the failure to respond to R-loop-associated DNA damage enhances chromosomal damage, which is already elevated in WS cells.

Finally, we tested whether chromosomal damage, enhanced upon chemical inhibition of ATM in WS cells (Figure 3B), was mitigated by suppressing R-loop formation. Our analysis showed that the frequency of chromosomal aberrations was significantly attenuated in WS cells lacking ATM pathway and treated with DRB following mild replication stress (Figure 10B).

Overall, these findings suggest that accumulation of R-loops contributes to genome instability upon Aph-induced replication perturbation in WRN-deficient cells.

## DISCUSSION

In this study, we proposed a novel role for WRN in preventing aberrant R-loop formation in human cells. We demonstrate that, in response to mild replication stress, WRN-deficient cells accumulate R-loops that impair replication fork progression. We also establish that R-loop accumulation greatly promotes ATM signalling activation due to transient DNA double-strand breaks (DSBs) generated by the XPG-dependent processing of RNA–DNA hybrids. Importantly, we show that ATM pathway is required for protecting WS cells from accumulating high levels of R-loop-associated genomic instability upon mild replication stress.

It is well-recognized that a considerable source of genome instability derives from collisions between replication and transcription complexes, which can be caused by formation of excessive transcription-associated structures known as R-loops (7). Moreover, recent studies have revealed that defects in fork protection factors play a crucial role in the onset of replication-transcription conflicts (21,61,62). WRN is a RecQ helicase whose primary function is maintaining genome integrity and ensure accurate handling of stalled replication forks (38,63). From this point of view, loss of WRN might hinder replication fork progression increasing the possibility that transcription machineries collide head-on with slowly-moving replication forks before transcription is terminated. Interestingly, although the helicase activity of WRN is required for efficient replication under replication stress (64), it is not involved in preventing R-loop accumulation. Some DNA helicases can promote fork protection by regulating R-loop metabolism without necessarily involving the helicase activity (17,19,65). Our results would suggest a similar enzymatic-independent function of WRN in counteracting unprogrammed R-loops under mild replication stress. Consistently, the helicase activity of WRN is not particularly active on RNA–DNA hybrids *in vitro* (66). Moreover, our data indicate that is the accumulation of R-loop to induce, at least partially, delayed replication in WRN-deficient cells and not *vice versa*. This is in line with previous demonstration that deregulated R-loops being a cause of replication fork stalling (67).

It has been reported that checkpoint response is required for the handling of R-loops (68–70). Defective early activation of CHK1 is observed in WRN-deficient cells, but not in cells expressing the helicase-dead WRN when replication is mildly inhibited (35). Interestingly, loss of WRN helicase activity does not compromise CHK1 phosphorylation (35), and does not result in a strong propensity to accumulate R-loops upon mild replication stress. Hence, it is reasonable that is the role of WRN in mediating ATR-checkpoint activation that counteracts the appearance of R-loops after Aph-induced replication perturbation. Several factors involved in removing R-loops, directly or indirectly, have been demonstrated to be regulated in a checkpoint-dependent manner, for instance, the Ddx19 RNA helicase (71). Ddx19 is implicated in removing R-loops derived from replication and transcription interference, and is specifically regulated by the ATR/CHK1 pathway (71). Thus, because of the inability of WS cells to activate the ATR/CHK1 signalling, Ddx19 could not be relocalized to nucleus and clearance of R-loops could be impeded (71). Similarly, some fork protection factors, such as FANCM and BLM, which participate in R-loop removal, could be crucial to restrain R-loop accumulation (16,18). Since FANCM and BLM are regulated by ATR (72,73), it is likely that, in WRN-deficient cells, they could be not activated due to impaired ATR signalling. However, given the complexity of this scenario, further studies will be needed to clarify the precise WRN function in R-loop metabolism.

Although WRN deficiency hampers replication checkpoint activation, CHK1 is phosphorylated upon prolonged exposure to low-dose of Aph (35). Interestingly, in the absence of WRN, late CHK1 phosphorylation is mostly ATM-dependent. ATM pathway is primarily activated in response to DSBs (47,58). However, previous studies have demonstrated that ATM responds to stimuli that do not produce DNA breakage such as Aph-induced mild replication stress (40,74). Moreover, a DSB-independent but R-loop-dependent ATM activation has been reported in quiescent cells (20). Although under our conditions we fail to detect DSBs, transcription inhibition or removal of R-loops reduces ATM phosphorylation and consistently CHK1 activation in WS cells. In contrast, WRN helicase-dead cells are not defective in CHK1 activation (35) neither accumulate R-loops. Consistently, they are not sensitive to inhibition of ATM or transcription, and do not elicit ATM pathway upon mild replication stress unless CHK1 is inhibited. This suggests that, in WS cells, there may be a link between R-loops and ATM signalling. Supporting this possibility, we obtained evidence that ATM associates with R-loops following Aph treatment, and that active transcription is required for the formation of PLA dots. Also, co-localization of pATM with pRPA, which correlates with R-loop formation (55), corroborates our hypothesis. Alternatively, ATM pathway could be triggered to repair DNA breaks deriving from R-loop-processing. Indeed, although we do not detect DSB formation, loss of ATM activity in WS cells results in a transcription-dependent DSB accumulation. Interestingly, we find a significant suppression of DSB formation in WS cells in which both XPG and ATM activities are abolished. The NER endonucleases XPG and XPF directly process aberrant RNA–DNA hybrids into DSBs (59). Under our conditions, transient XPG-mediated DSBs are formed in the absence of WRN leading to ATM signalling activation and DNA repair. Earlier study demonstrated that persistent DNA breaks induce an ATM-dependent silencing of transcription within the rDNA, the most actively transcribed region of the human genome, resulting in DDR activation (75). Similarly, in WRN-deficient cells ATM might be activated not only to stimulate repair but also to limit transcription, contributing to prevent massive DNA breakage.

In keeping with the notion that ATM and ATR play not redundant roles in human cells (76,77), ATM acts as a protective mechanism to limit the elevated genomic instability in WS cells. Furthermore, ATM activity is localized to sites of Aph-induced fork stalling. Hence, given that depletion of CHK1, but not CHK2, destabilizes common fragile sites (CFS) (78), it is plausible that ATM-dependent triggering of CHK1 is instrumental for counteracting instability at CFS, which is already elevated in WRN-deficient cells (35,36). Supporting this hypothesis bypassing WRN function in establishing the replication checkpoint through overexpression of a phospho-mimic form of CHK1 prevents ATM signalling upon mild replication stress. It is known that CFS are considered as hotspots of replication-transcription collisions (6). Loss of WRN or its helicase activity results in enhanced CFS instability (36). However, removal of R-loops after Aph-induced replication stress significantly suppresses chromosomal aberrations in WS cells, but not in WRN helicase-dead cells. These observations are consistent with a checkpoint-dependent and independent role of WRN in preserving genome integrity in response to mild replication stress, as proposed previously (35). Moreover, our findings suggest that R-loop accumulation could contribute significantly to genome instability in WS cells.

Hence, we propose that WRN exerts its function in maintenance of genome integrity through its potential role as regulator of conflicts arising between replication and transcription machineries. Loss of WRN leads to DSB-mediated ATM pathway activation in order to limit accumulation of R-loop-associated genomic instability upon mild replication stress (Figure 10). Because WRN deficiency is associated with cancer susceptibility (25), this study provides further evidence that replication-transcription collisions contribute to develop human disease and cancer.

## Supplementary Material

Supplementary Data
