# Supplementary material for: ATM pathway activation limits R-loop-associated genomic instability in Werner syndrome cells
Source: Nucleic Acids Res. 2019 Jan 18;47(7):3485–502. doi: 10.1093/nar/gkz025 (PMC6468170; doi:10.1093/nar/gkz025)
Supplement: Supplementary Data [file gkz025_supplemental_files.pdf]

SUPPLEMENTARY MATERIALS AND METHODS

Chemicals

Chemicals used were commercially obtained for the replication stress-inducing drug: aphidicolin (Sigma-Aldrich), for ATR inhibitor (VE-821; Selleckchem), ATM inhibitors (KU-55933 or KU-60019; Selleckchem), CHK1 inhibitor (UCN-01; Alexis Biochemicals) and the transcription elongation inhibitor: 5,6-dichloro-1-ß-D-ribofurosylbenzimidazole (DRB, Sigma-Aldrich). The final concentrations of the drugs used were: 0.4 µM aphidicolin, 5 µM VE-821, 10 µM KU-55933, 3 µM KU-60019, 100 nM UCN-01 and 50 µM DRB. Stock solutions for all of the pharmacological inhibitors were prepared in DMSO at a concentration of >1000-fold. The final concentration of DMSO in the culture medium was always < 0.1%.

Plasmids and RNA interference XPG

The phospho-mimic (Flag-CHK1$^{317D/345D}$) mutant form of CHK1, a kind gift from Prof. K.K. Kanna (Queensland Institute of Medical Research, Australia), was constructed as previously described (1). To express the plasmids, cells were transfected using the Neon™ Transfection System Kit (Invitrogen), according to the manufacturer’s instructions. The construct used to perform RNaseH1 overexpression experiments is a generous gift from Prof. R.J. Crouch (National Institutes of Health, Bethesda, USA). As previously described (2), the GFP-tagged RNaseH1 plasmid was generated by introducing a mutation on Met27 abrogating mitochondrial localization signal (RNaseH1-M27). To express the plasmids, cells were transfected using the Neon™ Transfection System Kit (Invitrogen), according to the manufacturer’s instructions.

XPG genetic knockdown experiments were performed by Interferin (Polyplus), according to the manufacturer’s instructions. siRNAs were used at 15 nM. As a control, a siRNA duplex directed against GFP was used. XPG depletion was achieved using siRNAs (QIAGEN) targeting the 3’UTR regions of proteins: XPG (5’- GAACGCACCUGCUGCUGUA-3’). Depletion of the proteins was confirmed by Western blot using an anti-XPG antibody (Santa Cruz Biotechnology, Inc.).

Western blot analysis

Cell lysates for Western blot were prepared as previously reported (3). Antibodies used for Western blot were commercially obtained for phospho-CHK1-Ser345 (Cell Signaling Technologies), CHK1 (Cell Signalling Technologies), phospho-ATM-Ser1981 (Millipore), ATM (Rockland), phospho-CHK2-Thr68 (Cell Signalling Technologies), CHK2 (Calbiochem), Flag M2 (Sigma-Aldrich), Cyclin A (Santa Cruz Biotechnology, Inc.), phospho-KAP1-Ser824 (Bethyl Laboratories), KAP1

(Bethyl Laboratories), XPG (Santa Cruz Biotechnology, Inc.), and GAPDH (Millipore). Horseradish peroxidase-conjugated goat specie-specific secondary antibodies (Santa Cruz Biotechnology, Inc.) were used. Quantification was performed on scanned images of blots using ImageJ software, and values are shown on the graphs as indicated.

Neutral and alkaline Comet assay

The occurrence of DNA double-strand breaks was evaluated by neutral Comet assay as described (4). Cell DNA was stained with a fluorescent dye GelRed (Biotium), and examined at 40× magnification with an Olympus fluorescence microscope. Slides were analysed by a computerized image analysis system (Comet IV, Perceptive UK). To assess the amount of DNA damage, computer-generated tail moment values (tail length × fraction of total DNA in the tail) were used. A minimum of 200 cells was analysed for each experimental point. Apoptotic cells (smaller comet head and extremely larger comet tail) were excluded from the analysis to avoid artificial enhancement of the tail moment.

DNA breakage induction was examined by alkaline Comet assay (single-cell gel electrophoresis) in denaturing conditions as described (5). Cell DNA was stained with a fluorescent dye GelRed (Biotium), and examined at 40× magnification with an Olympus fluorescence microscope. Slides were analysed as described above.

Chromosomal aberrations

Cells for metaphase preparations were collected according to standard procedure and as previously reported (6). Cell suspension was dropped onto cold, wet slides to make chromosome preparations. The slides were air dried overnight, then for each condition of treatment, the number of breaks and gaps was observed on Giemsa-stained metaphases. For each time point, at least 50 chromosome metaphases were examined by two independent investigators, and chromosomal damage was scored at 100× magnification with an Olympus fluorescence microscope.

Immunofluorescence for micronuclei

For micronuclei assay, cells were pre-treated or not with ATM inhibitor (KU-55933, 10 µM for 1 h), and then exposed or not to Aph (0.4 µM for 24 h). At the end of the treatment, cells were fixed in 2% formaldehyde for 10 min, and permeabilized using 0.4% Triton X-100 for 10 min. The fixed cells were washed with PBS, dehydrated with 70%, 90%, and 100% ethanol for three minutes each, and mounted in Vectashield containing 0.5 µg/ml DAPI. Cells were considered micronucleus-positive if they contained at least one micronucleus. For each time point and treatment, at least 400 cells were

scored as positive or negative for micronuclei. Images were scored at 40× magnification and captured using Eclipse 80i Nikon Fluorescence Microscope, equipped with a VideoConfocal (ViCo) system.

LIVE/DEAD assay

Viability was evaluated by the fluorescence-based assay the LIVE/DEAD Cell Double Staining Kit (Sigma-Aldrich), according to the manufacturer's instructions and as previously described (3). LIVE/DEAD assay is a short-term viability assay that allows direct evaluation of the number of live cells, stained in green with calcein-AM, and that of dead cells, stained in red with propidium iodide (PI). Since both calcein and PI-DNA can be excited with 490 nm light, simultaneous monitoring of live and dead cells is possible with a fluorescence microscope. Cell number was counted in randomly chosen fields and expressed as percent of dead cells (number of red nuclear stained cells/total cell number). For each time point, at least 1000 cells were counted.

***In situ* PLA assay**

The *in situ* proximity-ligation assay (PLA; Olink, Bioscience) was performed according to the manufacturer's instructions and as previously described (3). Briefly, antibody staining was carried out according to the standard immunofluorescence procedure. The primary antibodies used were: mouse-monoclonal anti-RNA-DNA hybrids, S9.6 (Kerafast), rabbit-polyclonal anti-ATM (Novus Biological) and rabbit-polyclonal anti-phopsho-RPA32 (pRPAS33; Bethyl laboratories). The negative control consisted of using only one primary antibody. Samples were incubated with secondary antibodies conjugated with PLA probes MINUS and PLUS: the PLA probe anti-mouse PLUS and anti-rabbit MINUS (OLINK Bioscience). The incubation with all antibodies was accomplished in a moist chamber for 1 h at 37°C. Next, the PLA probes MINUS and PLUS were ligated using two connecting oligonucleotides to produce a template for rolling-cycle amplification. After amplification, the products were hybridized with red fluorescence-labelled oligonucleotide. Samples were mounted in ProLong Gold antifade reagent with DAPI (blue). Images were acquired randomly using Eclipse 80i Nikon Fluorescence Microscope, equipped with a VideoConfocal (ViCo) system.

Cell cycle analysis by flow cytometry

Cells were treated with Aph 0.4 μM and harvested at the indicated times. Cells were processed for flow cytometry as described (3), and data analysed with CellQuest software.

SUPPLEMENTARY LEGENDS TO FIGURES

Figure S1. Loss of WRN results in ATM pathway activation upon mild replication stress. (A) Western blot of CHK1 activation in total extracts of Werner syndrome (WS) and WS corrected (WSWRN) cells treated as reported in the experimental design. Cells were exposed to ATMi (3 µM KU-60019) 1 h before to be treated or not with Aph. The presence of activated, i.e. phosphorylated, CHK1 was assessed using S345 phospho-specific antibody (pS345). Total amount of CHK1 was determined with anti-CHK1 antibody. Equal loading was confirmed probing the membrane with anti-GAPDH antibody. The fold increase with respect to the wild-type (WSWRN) untreated of the normalized ratio of the phosphorylated CHK1/total CHK1 is reported for each cell line. Representative gel images of at least two replicates are shown.

Figure S2. ATM inhibition does not enhance S-phase accumulation upon mild replication stress in WS cells. (A) Western blot detection of CHK1 activation in total extracts of Werner syndrome (WS) and WS corrected (WSWRN) cells, exposed or not to ATMi (KU-55933) 1 h prior to be treated or not with Aph. The presence of activated, i.e. phosphorylated, CHK1 was assessed using S345 phospho-specific antibody (pS345). Total amount of CHK1 was determined with anti-CHK1 antibody. Cyclin A was used to quantify S-phase cells. Equal loading was confirmed probing the membrane with anti-GAPDH antibody. The normalized ratio of the phosphorylated Cyclin A/GAPDH is given. Representative gel images of at least three replicates are shown.

Figure S3. Analysis of ATM pathway activation and cell cycle progression in WS cells upon short exposure times to mild replication stress. (A) Evaluation of ATM activation by immunofluorescence analysis in Werner syndrome (WS) and WS corrected (WSWRN) cells treated or not with Aph for the indicated times. Cells were fixed, and processed for immunostaining using a phosphospecific antibody raised against pATM (S1981). Dot plot shows data presented as pATM fluorescence intensity per nucleus from three independent experiments. Horizontal black lines represent the mean ± SE. Error bars represent standard error. (ns, not significant; *, $p < 0.1$; ****, $p < 0.0001$; two-tailed Student's t test). **(B)** Cell cycle progression analysis following mild replication stress. WSWRN or WS cells treated or not with Aph as in (A). Cells were harvested and stained with PI prior to FACS analysis. Graph shows the percentages of cells distributed into G_0/G_1, S or G_2/M phases.

Figure S4. ATM activation is not confined to S-phase in WS cells. Representative images showing Werner syndrome (WS) and WS corrected (WSWRN) cells treated or not with Aph, and co-immunostained with antibodies against pATM (S1981) and Cyclin A, a marker of S and G_2 phases. Nuclei were counterstained with DAPI. White arrows indicate Cyclin A positive cells.

Figure S5. CHK1 inhibition promotes ATM pathway activation in cells lacking the WRN helicase activity. Analysis of ATM activation by immunofluorescence analysis in Werner syndrome corrected (WSWRN) cells and WS cells expressing a mutant form of WRN affecting helicase function (WRN^{K577M}), exposed or not to Aph, and/or to UCN-01, a selective CHK1 inhibitor. Immunostaining was performed with an anti-pATM (S1981) antibody. Nuclei were counterstained with DAPI. Representative images of cells stained for pATM are given. Dot plot shows pATM intensity per nucleus from three independent experiments. Horizontal black lines represent the mean ± SE. (ns, not significant; ****, $p < 0.0001$; two-tailed Student's t test).

Figure S6. Expression of phospho-mimic CHK1 reduces CHK2 activation in WS cells. Western blot detection of CHK2 activation in total extracts of WSWRN or WS cells transfected with Flag-tagged $CHK1^{317/345D}$, and treated or not with Aph. The presence of activated, i.e. phosphorylated, CHK2 was assessed using T68 phospho-specific antibody (pT68CHK2). Total amount of CHK2 was determined with anti-CHK2 antibody. Expression levels of Flag-$CHK1^{317/345D}$ were determined by immunoblotting with anti-Flag antibody. Anti-GAPDH antibody was used to assess equal loading. The ratio of the phosphorylated CHK2/total CHK2 normalized to the untreated of each cell line is given. Representative gel images of at least three replicates are shown.

Figure S7. Inhibition of ATM enhances DNA damage in WRN-deficient cells upon mild replication perturbation. (A) Analysis of DNA damage accumulation evaluated by alkaline Comet assay. Werner syndrome (WS), WS corrected (WSWRN) cells and WRN helicase-defective (WRN^{K577M}) cells were used. Cells were exposed to ATMi (3 µM KU-60019) 1 h before to be treated or not with Aph as reported in the scheme, and then subjected to Comet assay. Graph shows data presented as mean tail moment ± SE from three independent experiments. Horizontal black lines represent the mean. (ns, not significant; ***, $p < 0.001$; ****, $p < 0.0001$; two-tailed Student's t test).

Figure S8. ATM inhibition in WS cells results in DSB formation upon mild replication stress. Analysis of DSB accumulation in Werner syndrome (WS) and WS corrected (WSWRN) cells. Cells were exposed or not to ATMi (KU-55933) 1 h prior to be treated or not with Aph, then subjected to

neutral Comet assay. Representative images are shown. Dot plot shows data presented as mean tail moment ± SE from three independent experiments. Horizontal black lines represent the mean. (ns, not significant; ****, $p < 0.0001$; two-tailed Student's t test).

Figure S9. Inhibition of ATM enhances chromosomal damage in WS cells upon mild replication stress. Representative images from the analysis of chromosomal aberrations in Werner syndrome (WS) cells, WS corrected (WSWRN) cells and WS cells expressing a mutant form of WRN affecting helicase function (WRN^{K577M}), treated or not with ATMi 1h prior to be exposed to Aph. Metaphases were collected by colcemid. Next, cells were fixed and processed as reported in "Supplementary Materials and Methods". Red arrows indicate chromosomal aberrations. Insets show enlarged metaphases for a better evaluation of chromosomal aberrations.

Figure S10. Inhibition of ATM increases DNA damage and cell death in WRN-deficient cells upon mild replication perturbation. (A) Evaluation of micronuclei formation in WSWRN, WS and WRN^{K577M} cells treated as in the experimental design. Nuclei were counterstained with DAPI. Representative images are given. Bar graph shows the percentage of cells with micronuclei from three independent experiments ± SE from three independent experiments. (ns, not significant; **, $p < 0.01$; two-tailed Student's t test). White arrows indicate micronuclei. **(B)** Evaluation of cell death in WSWRN, WS and WRN^{K577M} cells treated as reported in the scheme. Cell viability was evaluated by the fluorescence-based LIVE/DEAD assay as described in "Supplementary Materials and Methods". Representative images of double-staining of viable (green) and dead (red) cells are shown. Data are expressed as mean of dead cells ± SE from three independent experiments. (ns, not significant; *, $p < 0.1$; ***, $p < 0,001$; two-tailed Student's t test).

Figure S11. Evaluation of cell cycle progression following transcription inhibition. Werner syndrome (WS) or WS corrected (WSWRN) cells were exposed or not to transcription inhibitor (DRB) for 3 h. Cells were harvested and stained with PI prior to FACS analysis. Graph shows the percentages of cells distributed into G_0/G_1, S or G_2/M phases.

Figure S12. Transcription inhibition suppresses ATM hyperactivation in WS cells upon mild replication stress. Evaluation of ATM activation by immunofluorescence analysis in Werner syndrome (WS), WS corrected (WSWRN) cells and WS cells expressing a mutant form of WRN affecting helicase function (WRN^{K577M}), treated as reported in the experimental design and exposed or not to transcription inhibitor (DRB). Immunostaining was performed with anti-pATM (S1981)

antibody. Dot plot shows pATM intensity per nucleus from three independent experiments. Horizontal black lines represent the mean ± SE. (****, $p < 0.0001$; two-tailed Student's t test).

Figure S13. Phospho-ATM colocalizes at R-loops in WS cells upon mild replication stress. Evaluation of the percentage of pATM-positive cells colocalising or not with pRPA foci in Werner syndrome (WS) and WS corrected (WSWRN) cells, treated or not with Aph. Cells were co-immunostained with antibodies against pATM (S1981) and pRPA32 (S33). Nuclei were counterstained with DAPI. Representative images are given. Bar graph shows the percentage of pATM positive cells in pRPA-positive ($pATM^+/pRPA^+$) or negative ($pATM^+/pRPA^-$) cells from three independent experiments. Horizontal black lines represent the mean ± SE. (****, $p < 0.0001$; two-tailed Student's t test).

Figure S14. Analysis of RNaseH1 overexpression in the cells. Werner syndrome (WS) cells, WS corrected (WSWRN) cells and WS cells expressing a mutant form of WRN affecting helicase function (WRN^{K577M}) were transfected with GFP-tagged RNaseH1 construct. Expression levels of transfected GFP-RNase H1 were determined by fluorescence microscopy. Representative images are given and the percentage of GFP positive cells (green) are reported. Nuclei were counterstained with DAPI (blue).

REFERENCES

1. Gatei,M., Sloper,K., Sörensen,C., Syljuäsen,R., Falck,J., Hobson,K., Savage,K., Lukas,J., Zhou,B.-B., Bartek,J., *et al.* (2003) Ataxia-telangiectasia-mutated (ATM) and NBS1-dependent Phosphorylation of Chk1 on Ser-317 in Response to Ionizing Radiation. *J. Biol. Chem.*, **278**, 14806–14811.
2. Cerritelli,S.M., Frolova,E.G., Feng,C., Grinberg,A., Love,P.E. and Crouch,R.J. (2003) Failure to produce mitochondrial DNA results in embryonic lethality in Rnaseh1 null mice. *Mol. Cell*, **11**, 807–815.
3. Leuzzi,G., Marabitti,V., Pichierri,P. and Franchitto,A. (2016) WRNIP 1 protects stalled forks from degradation and promotes fork restart after replication stress. *EMBO J.*, **35**, 1–15.
4. Murfuni,I., De Santis,A., Federico,M., Bignami,M., Pichierri,P. and Franchitto,A. (2012) Perturbed replication induced genome wide or at common fragile sites is differently managed in the absence of WRN. *Carcinogenesis*, **33**, 1655–1663.

5. Pichierri,P., Franchitto,A., Mosesso,P., Palitti,F. and Molecolare,C. (2001) Werner ’ s Syndrome Protein Is Required for Correct Recovery after Replication Arrest and DNA Damage Induced in S-Phase of Cell Cycle. *Mol. Biol. Cell*, **12**, 2412–2421.
6. Pirzio,L.M., Pichierri,P., Bignami,M. and Franchitto,A. (2008) Werner syndrome helicase activity is essential in maintaining fragile site stability. *J. Cell Biol.*, **180**, 305–314.

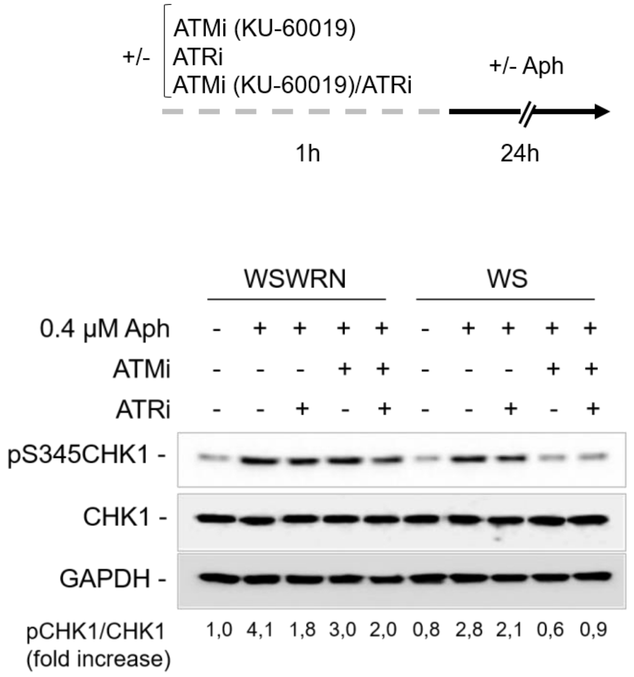

Suppl. Figure 1

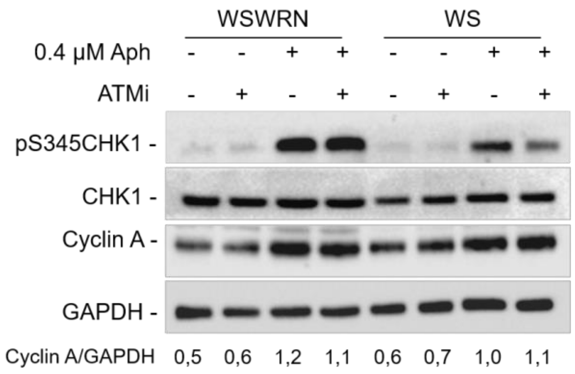

Suppl. Figure 2

A

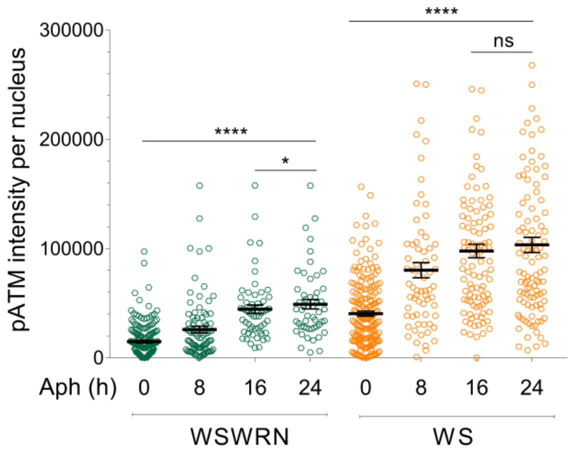

B

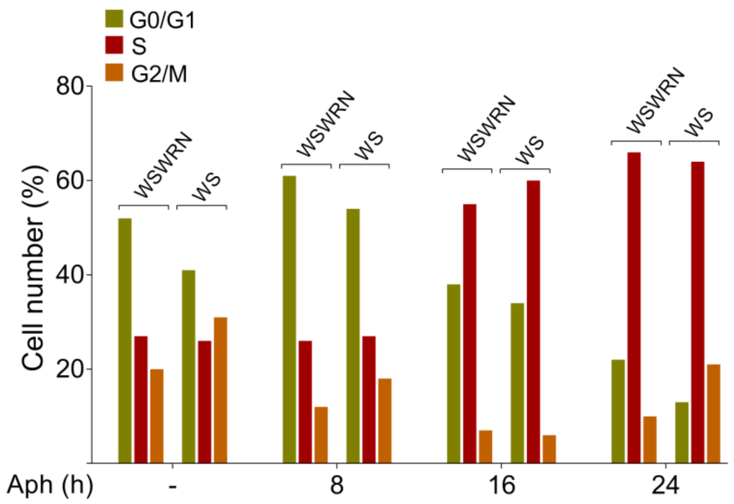

Suppl. Figure 3

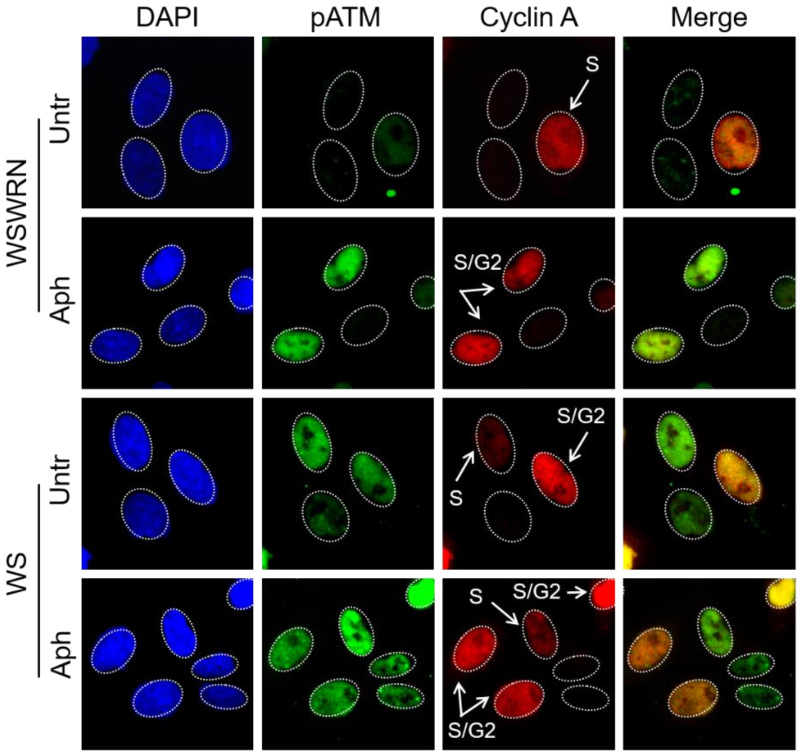

Suppl. Figure 4

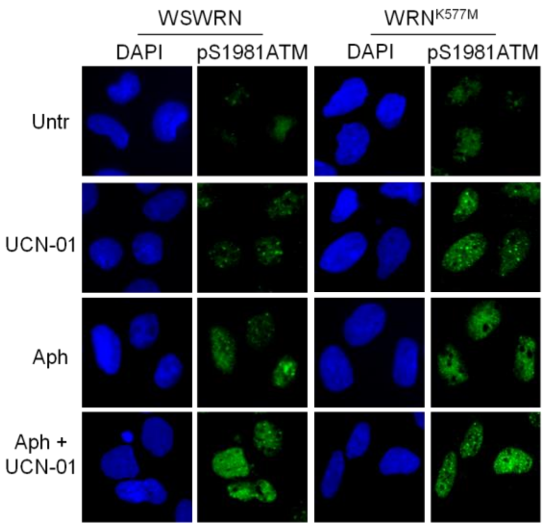

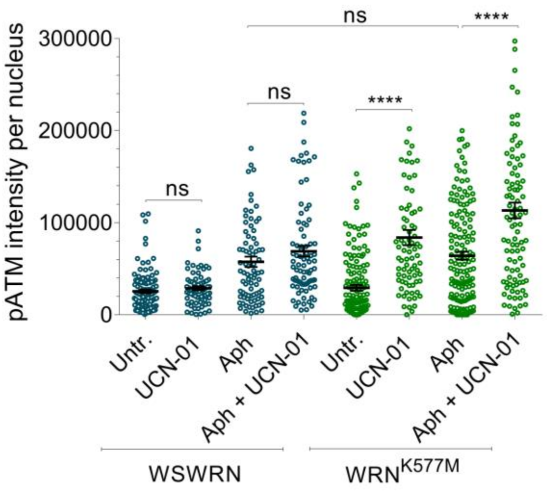

Suppl. Figure 5

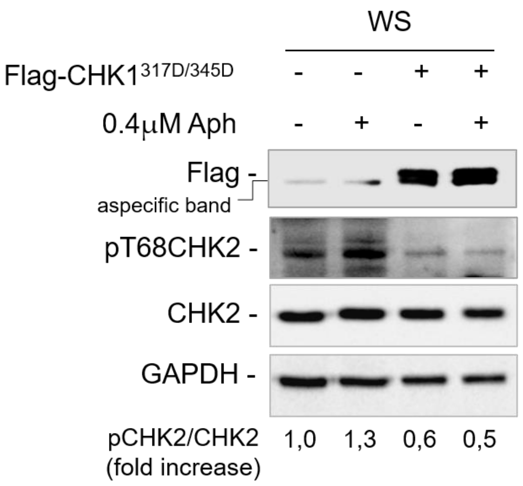

Suppl. Figure 6

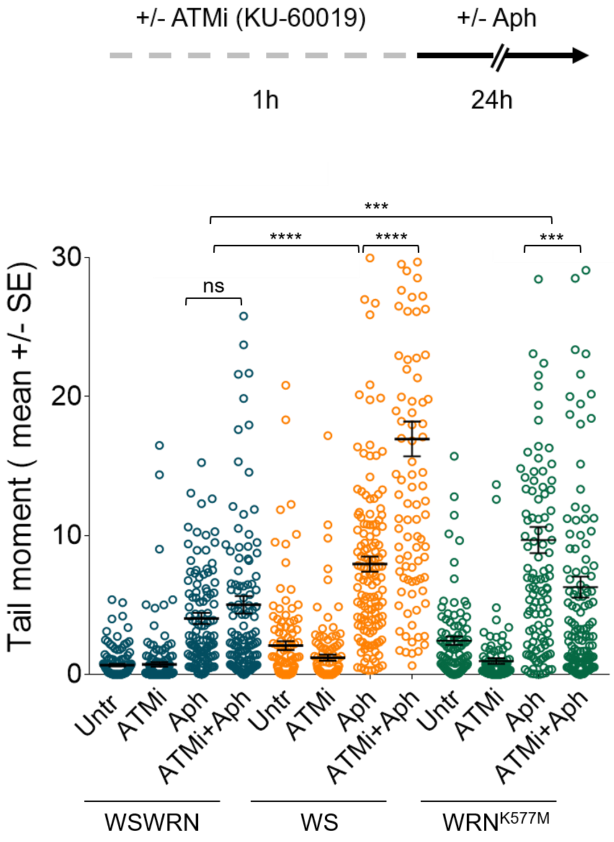

Suppl. Figure 7

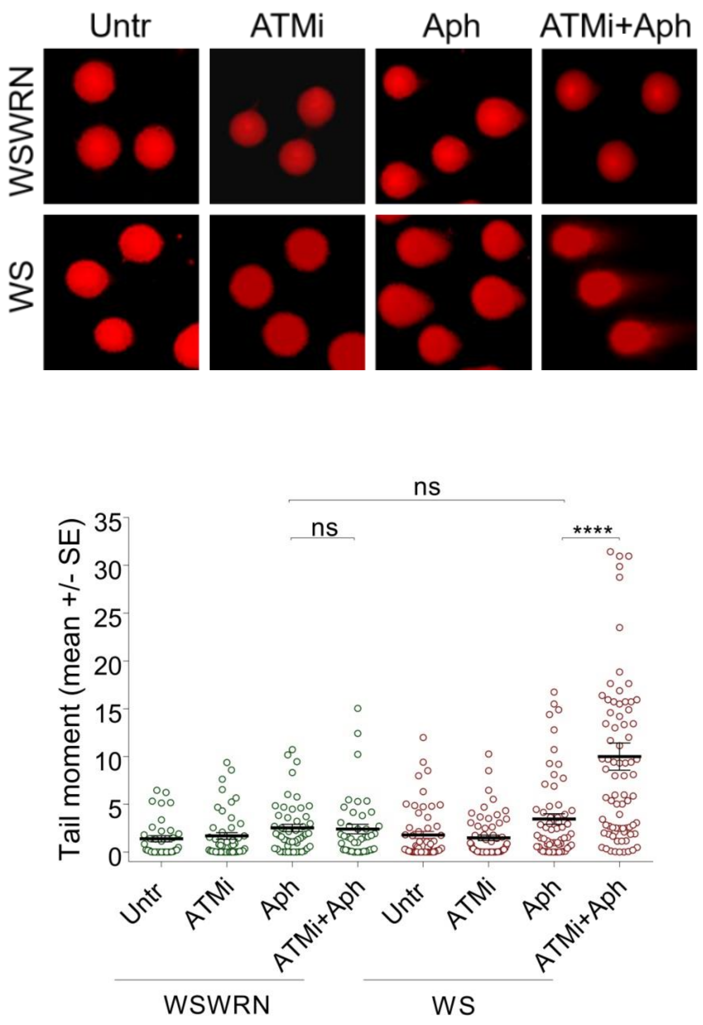

Suppl. Figure 8

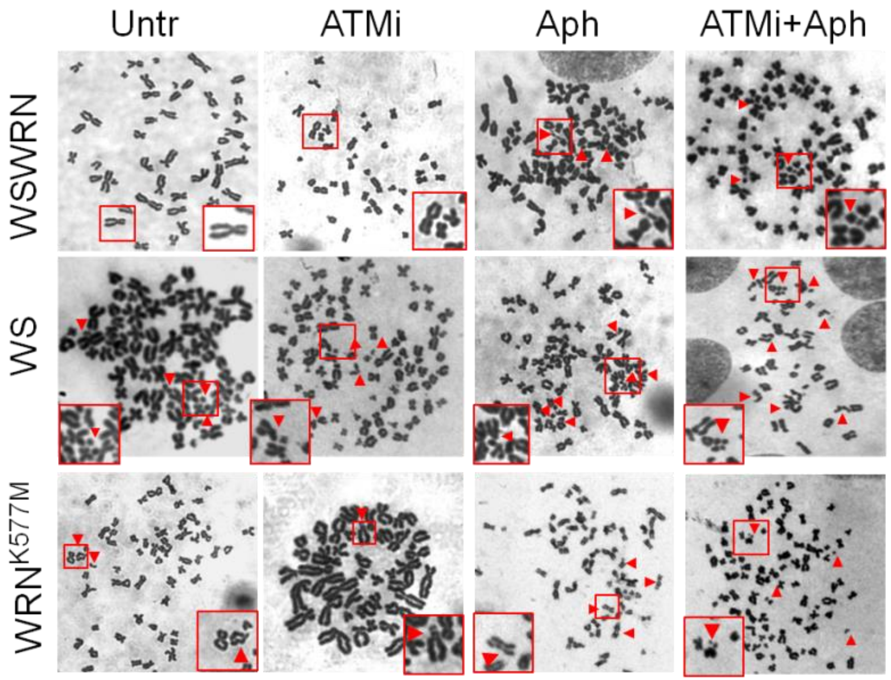

Suppl. Figure 9

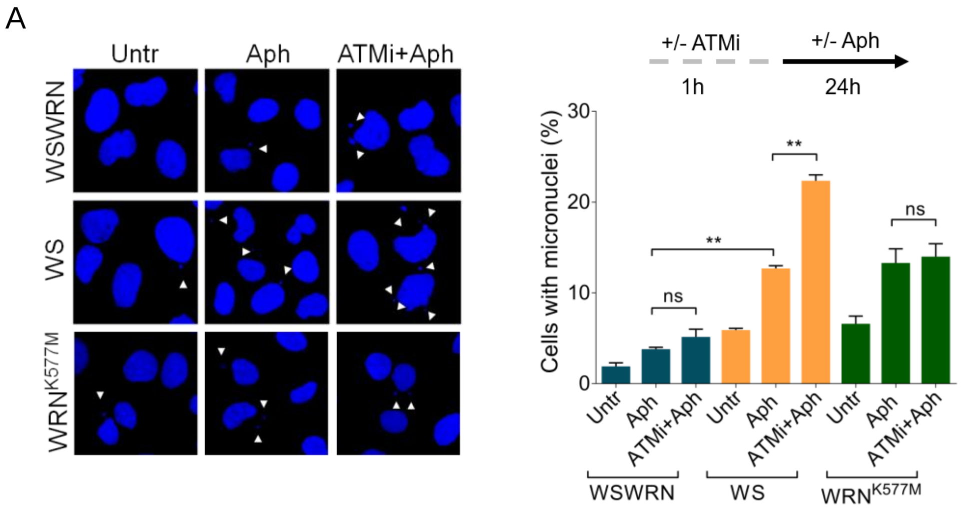

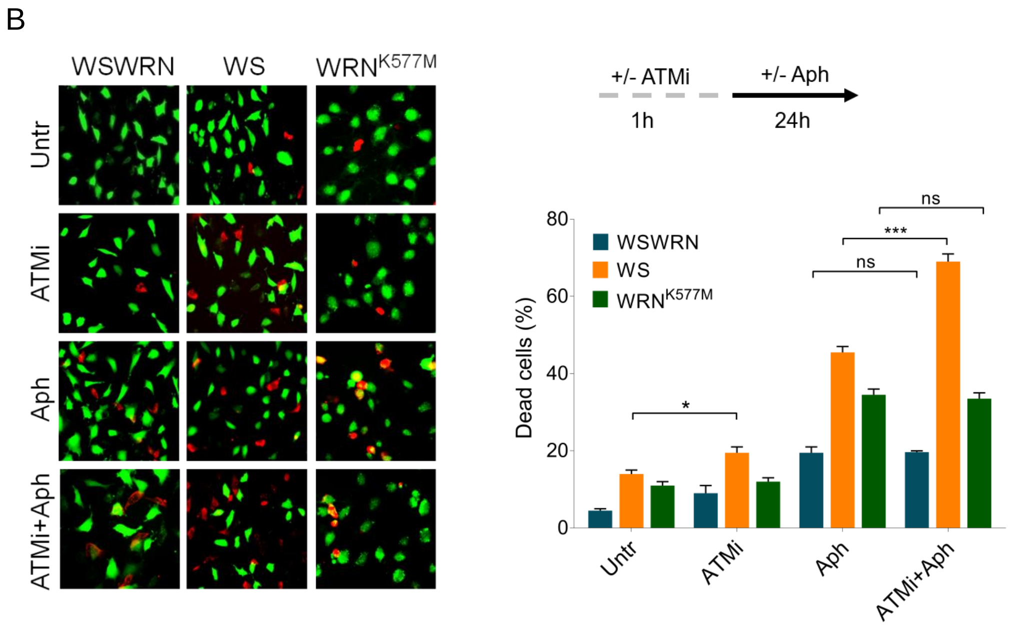

Suppl. Figure 10

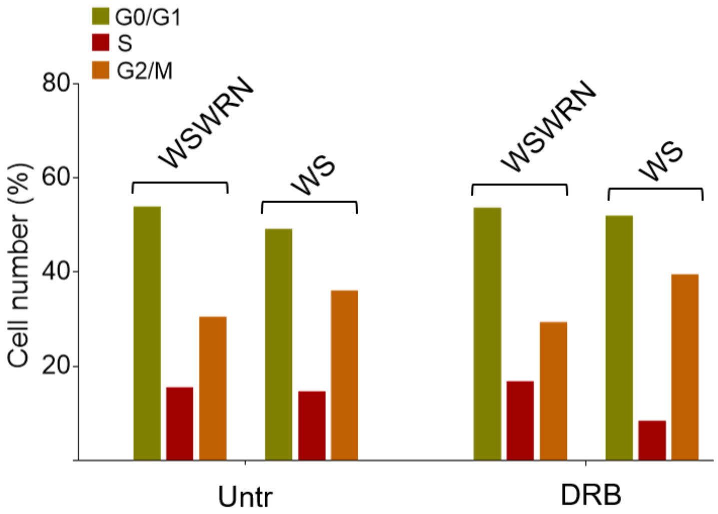

Suppl. Figure 11

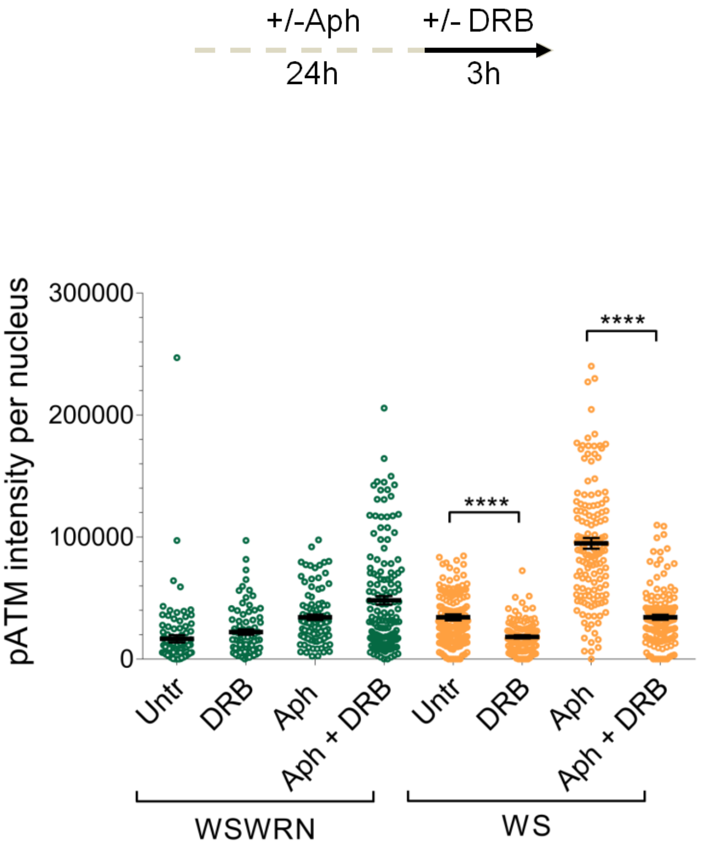

Suppl. Figure 12

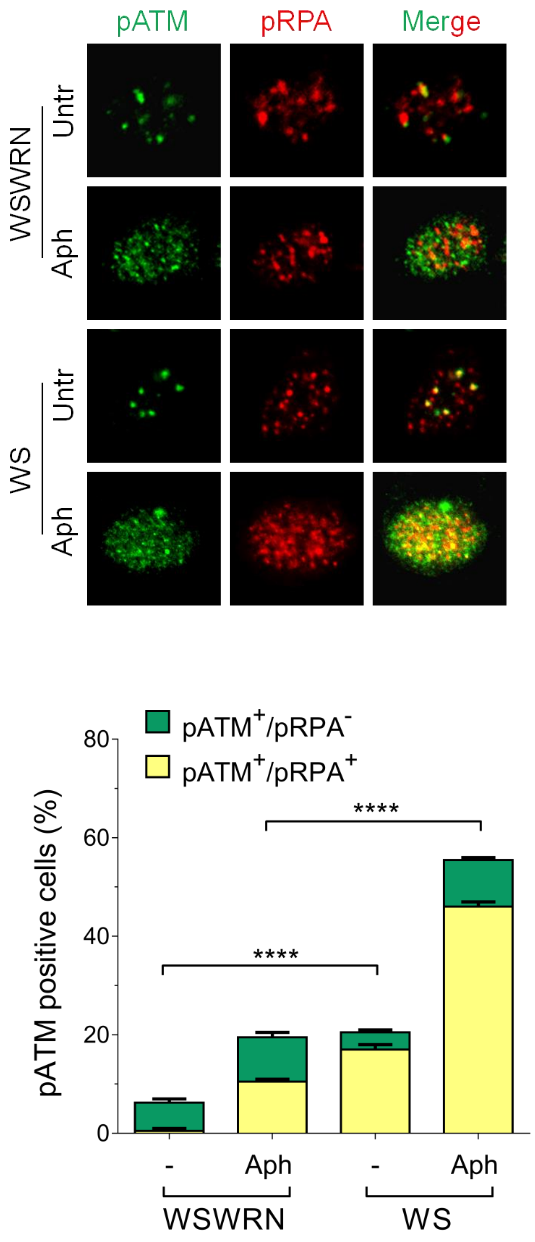

Suppl. Figure 13

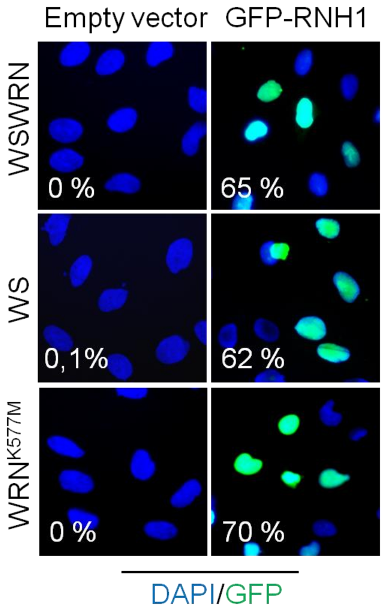

Suppl. Figure 14
